# The PREDICTS database: a global database of how local terrestrial biodiversity responds to human impacts

**DOI:** 10.1002/ece3.1303

**Published:** 2014-12-02

**Authors:** Lawrence N. Hudson, Tim Newbold, Sara Contu, Samantha L. L. Hill, Igor Lysenko, Adriana De Palma, Helen R. P. Phillips, Rebecca A. Senior, Dominic J. Bennett, Hollie Booth, Argyrios Choimes, David L. P. Correia, Julie Day, Susy Echeverría‐Londoño, Morgan Garon, Michelle L. K. Harrison, Daniel J. Ingram, Martin Jung, Victoria Kemp, Lucinda Kirkpatrick, Callum D. Martin, Yuan Pan, Hannah J. White, Job Aben, Stefan Abrahamczyk, Gilbert B. Adum, Virginia Aguilar‐Barquero, Marcelo A. Aizen, Marc Ancrenaz, Enrique Arbeláez‐Cortés, Inge Armbrecht, Badrul Azhar, Adrián B. Azpiroz, Lander Baeten, András Báldi, John E. Banks, Jos Barlow, Péter Batáry, Adam J. Bates, Erin M. Bayne, Pedro Beja, Åke Berg, Nicholas J. Berry, Jake E. Bicknell, Jochen H. Bihn, Katrin Böhning‐Gaese, Teun Boekhout, Céline Boutin, Jérémy Bouyer, Francis Q. Brearley, Isabel Brito, Jörg Brunet, Grzegorz Buczkowski, Erika Buscardo, Jimmy Cabra‐García, María Calviño‐Cancela, Sydney A. Cameron, Eliana M. Cancello, Tiago F. Carrijo, Anelena L. Carvalho, Helena Castro, Alejandro A. Castro‐Luna, Rolando Cerda, Alexis Cerezo, Matthieu Chauvat, Frank M. Clarke, Daniel F. R. Cleary, Stuart P. Connop, Biagio D'Aniello, Pedro Giovâni da Silva, Ben Darvill, Jens Dauber, Alain Dejean, Tim Diekötter, Yamileth Dominguez‐Haydar, Carsten F. Dormann, Bertrand Dumont, Simon G. Dures, Mats Dynesius, Lars Edenius, Zoltán Elek, Martin H. Entling, Nina Farwig, Tom M. Fayle, Antonio Felicioli, Annika M. Felton, Gentile F. Ficetola, Bruno K. C. Filgueiras, Steven J. Fonte, Lauchlan H. Fraser, Daisuke Fukuda, Dario Furlani, Jörg U. Ganzhorn, Jenni G. Garden, Carla Gheler‐Costa, Paolo Giordani, Simonetta Giordano, Marco S. Gottschalk, Dave Goulson, Aaron D. Gove, James Grogan, Mick E. Hanley, Thor Hanson, Nor R. Hashim, Joseph E. Hawes, Christian Hébert, Alvin J. Helden, John‐André Henden, Lionel Hernández, Felix Herzog, Diego Higuera‐Diaz, Branko Hilje, Finbarr G. Horgan, Roland Horváth, Kristoffer Hylander, Paola Isaacs‐Cubides, Masahiro Ishitani, Carmen T. Jacobs, Víctor J. Jaramillo, Birgit Jauker, Mats Jonsell, Thomas S. Jung, Vena Kapoor, Vassiliki Kati, Eric Katovai, Michael Kessler, Eva Knop, Annette Kolb, Ádám Kőrösi, Thibault Lachat, Victoria Lantschner, Violette Le Féon, Gretchen LeBuhn, Jean‐Philippe Légaré, Susan G. Letcher, Nick A. Littlewood, Carlos A. López‐Quintero, Mounir Louhaichi, Gabor L. Lövei, Manuel Esteban Lucas‐Borja, Victor H. Luja, Kaoru Maeto, Tibor Magura, Neil Aldrin Mallari, Erika Marin‐Spiotta, E. J. P. Marshall, Eliana Martínez, Margaret M. Mayfield, Grzegorz Mikusinski, Jeffrey C. Milder, James R. Miller, Carolina L. Morales, Mary N. Muchane, Muchai Muchane, Robin Naidoo, Akihiro Nakamura, Shoji Naoe, Guiomar Nates‐Parra, Dario A. Navarrete Gutierrez, Eike L. Neuschulz, Norbertas Noreika, Olivia Norfolk, Jorge Ari Noriega, Nicole M. Nöske, Niall O'Dea, William Oduro, Caleb Ofori‐Boateng, Chris O. Oke, Lynne M. Osgathorpe, Juan Paritsis, Alejandro Parra‐H, Nicolás Pelegrin, Carlos A. Peres, Anna S. Persson, Theodora Petanidou, Ben Phalan, T. Keith Philips, Katja Poveda, Eileen F. Power, Steven J. Presley, Vânia Proença, Marino Quaranta, Carolina Quintero, Nicola A. Redpath‐Downing, J. Leighton Reid, Yana T. Reis, Danilo B. Ribeiro, Barbara A. Richardson, Michael J. Richardson, Carolina A. Robles, Jörg Römbke, Luz Piedad Romero‐Duque, Loreta Rosselli, Stephen J. Rossiter, T'ai H. Roulston, Laurent Rousseau, Jonathan P. Sadler, Szabolcs Sáfián, Romeo A. Saldaña‐Vázquez, Ulrika Samnegård, Christof Schüepp, Oliver Schweiger, Jodi L. Sedlock, Ghazala Shahabuddin, Douglas Sheil, Fernando A. B. Silva, Eleanor M. Slade, Allan H. Smith‐Pardo, Navjot S. Sodhi, Eduardo J. Somarriba, Ramón A. Sosa, Jane C. Stout, Matthew J. Struebig, Yik‐Hei Sung, Caragh G. Threlfall, Rebecca Tonietto, Béla Tóthmérész, Teja Tscharntke, Edgar C. Turner, Jason M. Tylianakis, Adam J. Vanbergen, Kiril Vassilev, Hans A. F. Verboven, Carlos H. Vergara, Pablo M. Vergara, Jort Verhulst, Tony R. Walker, Yanping Wang, James I. Watling, Konstans Wells, Christopher D. Williams, Michael R. Willig, John C. Z. Woinarski, Jan H. D. Wolf, Ben A. Woodcock, Douglas W. Yu, Andrey S. Zaitsev, Ben Collen, Rob M. Ewers, Georgina M. Mace, Drew W. Purves, Jörn P. W. Scharlemann, Andy Purvis

**Affiliations:** ^1^ Department of Life Sciences Natural History Museum Cromwell Road London SW7 5BD U.K; ^2^ United Nations Environment Programme World Conservation Monitoring Centre 219 Huntingdon Road Cambridge CB3 0DL U.K; ^3^ Computational Ecology and Environmental Science Microsoft Research 21 Station Road Cambridge CB1 2FB U.K; ^4^ Imperial College London Silwood Park Campus Buckhurst Road Ascot SL5 7PY U.K; ^5^ Frankfurt Zoological Society Africa Regional Office PO Box 14935 Arusha Tanzania; ^6^ School of Life Sciences University of Sussex Brighton BN1 9QG U.K; ^7^ Center for Macroecology, Climate and Evolution the Natural History Museum of Denmark Universitetsparken 15 2100 Copenhagen Denmark; ^8^ School of Biological and Ecological Sciences University of Stirling Bridge of Allan Stirling FK9 4LA U.K; ^9^ Department of Animal and Plant Sciences University of Sheffield Alfred Denny Building Western Bank Sheffield S10 2TN U.K; ^10^ School of Biological Sciences Queen's University Belfast 97 Lisburn Road Belfast BT9 7BL U.K; ^11^ Evolutionary Ecology Group University of Antwerp Groenenborgerlaan 171 2020 Antwerp Belgium; ^12^ Nees Institute for Plant Biodiversity University of Bonn Meckenheimer Allee 170 53113 Bonn Germany; ^13^ Department of Wildlife and Range Management FRNR, CANR, KNUST Kumasi Ghana; ^14^ SAVE THE FROGS! Ghana Box KS 15924 Adum‐Kumasi Ghana; ^15^ Escuela de Biología Universidad de Costa Rica 2060 San José Costa Rica; ^16^ CONICET, Lab. INIBIOMA (Universidad Nacional del Comahue‐CONICET) Pasaje Gutierrez 1125 8400 Bariloche Rio Negro Argentina; ^17^ HUTAN – Kinabatangan Orang‐utan Conservation Programme PO Box 17793 88874 Kota Kinabalu Sabah Malaysia; ^18^ Museo de Zoología, Facultad de Ciencias Universidad Nacional Autónoma de México México D.F Mexico; ^19^ Colección de Tejidos Instituto de Investigación de Recursos Biológicos Alexander von Humboldt Km 17 Cali‐Palmira Valle del Cauca Colombia; ^20^ Department of Biology Universidad del Valle Calle 13 #100‐00 Cali Colombia; ^21^ Biodiversity Unit, Institute of Bioscience Universiti Putra Malaysia 43400 Serdang Selangor Malaysia; ^22^ Faculty of Forestry Universiti Putra Malaysia 43400 Serdang Selangor Malaysia; ^23^ Laboratorio de Genética de la Conservación Instituto de Investigaciones Biológicas Clemente Estable Montevideo Uruguay; ^24^ Department of Forest and Water Management Forest & Nature Lab Ghent University Geraardsbergsesteenweg 267 9090 Gontrode Belgium; ^25^ Terrestrial Ecology Unit Department of Biology Ghent University K. L. Ledeganckstraat 35 9000 Gent Belgium; ^26^ MTA Centre for Ecological Research Alkotmány u. 2‐4 2163 Vácrátót Hungary; ^27^ University of Washington 1900 Commerce Street Tacoma Washington 98402 U.K; ^28^ Lancaster Environment Centre Lancaster University Lancaster LA1 4YQ U.K; ^29^ MCT/Museu Paraense Emílio Goeldi Belém Pará Brazil; ^30^ Agroecology Georg‐August University Grisebachstrasse 6 37077 Göttingen Germany; ^31^ University of Birmingham Edgbaston Birmingham B15 2TT U.K; ^32^ Department of Biological Sciences University of Alberta CW 405 – Biological Sciences Centre Edmonton AB T6G 2E9 Canada; ^33^ EDP Biodiversity Chair CIBIO/InBio, Centro de Investigação em Biodiversidade e Recursos Genéticos Universidade do Porto Campus Agrário de Vairão 4485‐601 Vairão Portugal; ^34^ The Swedish University of Agricultural Sciences The Swedish Biodiversity Centre SE 750 07 Uppsala Sweden; ^35^ University of Edinburgh School of GeoSciences Crew Building King's Buildings West Mains Road Edinburgh EH9 3JN U.K; ^36^ Durrell Institute of Conservation and Ecology (DICE) School of Anthropology and Conservation University of Kent Canterbury CT2 7NR U.K; ^37^ Iwokrama International Centre for Rainforest Conservation and Development 77 High Street Georgetown Guyana; ^38^ Department of Animal Ecology Philipps‐University Marburg Karl‐von‐Frisch Strasse 8 35032 Marburg Germany; ^39^ Biodiversity and Climate Research Centre (BiK‐F) Senckenberg Gesellschaft für Naturforschung Senckenberganlage 25 60325 Frankfurt am Main Germany; ^40^ Institute for Ecology, Evolution & Diversity Biologicum Goethe University Frankfurt Max von Laue St. 13 D 60439 Frankfurt am Main Germany; ^41^ CBS‐KNAW Fungal Biodiversity Centre Utrecht The Netherlands; ^42^ Environment Canada, Science & Technology Branch Carleton University 1125 Colonel By Drive Raven Road Ottawa ON K1A 0H3 Canada; ^43^ Unité Mixte de Recherche Contrôle des Maladies Animales Exotiques et Emergentes Centre de Coopération Internationale en Recherche Agronomique pour le Développement (CIRAD) 34398 Montpellier France; ^44^ Unité Mixte de Recherche 1309 Contrôle des Maladies Animales Exotiques et Emergentes Institut national de la recherche agronomique (INRA) 34398 Montpellier France; ^45^ School of Science and the Environment Manchester Metropolitan University Chester Street Manchester M1 5GD U.K; ^46^ University of Évora – ICAAMA, Apartado 94 7002‐554 Évora Portugal; ^47^ Southern Swedish Forest Research Centre Swedish University of Agricultural Sciences Box 49 230 53 Alnarp Sweden; ^48^ Department of Entomology Purdue University 901 W. State Street West Lafayette 47907 Indiana Portugal; ^49^ Centro de Ecologia Funcional Departamento de Ciências da Vida Universidade de Coimbra Calçada Martim de Freitas 3000‐456 Coimbra Portugal; ^50^ Escritório Central do LBA Instituto Nacional de Pesquisa da Amazônia Av. André Araújo, 2936, Campus II, Aleixo CEP 69060‐001 Manaus AM Brazil; ^51^ Department of Botany School of Natural Sciences Trinity College Dublin College Green Dublin 2 Ireland; ^52^ Departamento de Zoologia Instituto de Biociências Universidade de São Paulo São Paulo SP 05508‐090 Brazil; ^53^ Department of Ecology and Animal Biology Faculty of Sciences University of Vigo 36310 Vigo Spain; ^54^ Department of Entomology University of Illinois Urbana Illinois 61801 Brazil; ^55^ Museu de Zoologia da Universidade de São Paulo Av. Nazaré 481 04263‐000 São Paulo SP Brazil; ^56^ Instituto Nacional de Pesquisas da Amazônia Av. André Araújo, 2.936, Petrópolis CEP 69067‐375 Manaus AM Brazil; ^57^ Centre for Functional Ecology Department of Life Sciences University of Coimbra Calçada Martim de Freitas 3000‐456 Coimbra Portugal; ^58^ Instituto de Biotecnologia y Ecologia Aplicada (INBIOTECA) Universidad Veracruzana Av. de las Culturas Veracruzanas, 101, Col. Emiliano Zapata CP 91090 Xalapa Veracruz Mexico; ^59^ Centro Agronómico Tropical de Investigación y Enseñanza (CATIE) Tropical Agricultural Research and Higher Education Center 7170 Cartago Turrialba, 30501 Costa Rica; ^60^ Department of Quantitative Methods and Information Systems Faculty of Agronomy University of Buenos Aires Av. San Martín 4453 Ciudad Autónoma de Buenos Aires Argentina C.P. 1417 Argentina; ^61^ Normandie Univ. EA 1293 ECODIV‐Rouen SFR SCALE, UFR Sciences et Techniques 76821 Mont Saint Aignan Cedex France; ^62^ University of Aberdeen Aberdeen AB24 2TZ U.K; ^63^ Department of Biology CESAM Universidade de Aveiro Campus Universitário de Santiago 3810‐193 Aveiro Portugal; ^64^ Sustainability Research Institute University of East London 4‐6 University Way London E16 2RD U.K; ^65^ Department of Biology University of Naples “Federico II” Naples Italy; ^66^ Programa de Pós‐graduação em Ecologia Universidade Federal de Santa Catarina Florianópolis Santa Catarina CEP 88040‐900 Brazil; ^67^ British Trust for Ornithology University of Stirling Stirling FK9 4LA U.K; ^68^ Thünen Institute of Biodiversity Bundesallee 50 38116 Braunschweig Germany; ^69^ CNRS, Écologie des Forêts de Guyane (UMR‐CNRS 8172) BP 316 97379 Kourou cedex France; ^70^ Université de Toulouse UPS, INP, Laboratoire Écologie Fonctionnelle et Environnement (Ecolab) 118 route de Narbonne 31062 Toulouse France; ^71^ Department of Landscape Ecology Institute for Nature and Resource Conservation Kiel University Olshausenstrasse 75 24098 Kiel Germany; ^72^ Department of Biology Nature Conservation University Marburg Marburg Germany; ^73^ Institute of Integrative Biology ETH Zurich Switzerland; ^74^ Programa de Biología Universidad del Atlántico Km 7 vía Puerto Colombia Atlántico Colombia; ^75^ Biometry and Environmental System Analysis University of Freiburg Tennenbacher Strasse 4 79106 Freiburg Germany; ^76^ INRA, UMR1213 Herbivores 63122 Saint‐Genès‐Champanelle France; ^77^ Institute of Zoology Zoological Society of London Nuffield Building Regents Park London, NW1 4RY U.K; ^78^ Department of Ecology and Environmental Science Umeå University 901 87 Umeå Sweden; ^79^ Wildlife, Fish and Environmental Studies Swedish University of Agricultural Sciences 901 83 Umeå Sweden; ^80^ MTA‐ELTE‐MTM Ecology Research Group Hungarian Academy of Sciences, c/o Biological Institute Eötvös Lóránd University Pázmány Péter sétány 1/C. 1117 Budapest Hungary; ^81^ University of Koblenz‐Landau Institute for Environmental Sciences Fortstr. 7 76829 Landau Germany; ^82^ Department of Ecology – Conservation Ecology Faculty of Biology Philipps‐Universität Marburg Karl‐von‐Frisch‐Street 8 35032 Marburg Germany; ^83^ Faculty of Science University of South Bohemia and Institute of Entomology Biology Centre of Academy of Sciences Czech Republic Branišovská 31 370 05 České Budějovice Czech Republic; ^84^ Institute for Tropical Biology and Conservation Universiti Malaysia Sabah 88999 Kota Kinabalu Sabah Malaysia; ^85^ Dipartimento di Scienze Veterinarie Università di Pisa Viale delle Piagge, n°2 56124 Pisa Italy; ^86^ The Southern Swedish Forest Research Centre The Swedish University of Agricultural Sciences PO Box 49 23453 Alnarp Sweden; ^87^ Laboratoire d'Ecologie Alpine (LECA) Université Grenoble Alpes F‐38000 Grenoble France; ^88^ Programa de Pós‐Graduação em Biologia Animal Universidade Federal de Pernambuco Recife PE 50670‐901 Brazil; ^89^ Department of Plant Sciences University of California Davis California 95616 Canada; ^90^ Department of Natural Resource Sciences Thompson Rivers University 900 McGill Road Kamloops BC V2C 0C8 Canada; ^91^ IDEA Consultants Inc Okinawa Branch Office Aja 2‐6‐19 Naha Okinawa 900‐0003 Japan; ^92^ Carl Zeiss Microscopy GmbH Königsallee 9 – 21 37081 Göttingen Germany; ^93^ University of Hamburg Biocentre Grindel Martin‐Luther‐King Platz 3 20146 Hamburg Germany; ^94^ Seed Consulting Services 106 Gilles Street Adelaide 5000 SA Australia; ^95^ School of Geography Planning and Environmental Management The University of Queensland St Lucia 4072 Qld Australia; ^96^ Ecologia Aplicada/Applied Ecology Universidade Sagrado Coração (USC) Rua Irmã Arminda, 10‐50 Jardim Brasil Bauru São Paulo Brazil; ^97^ DISTAV University of Genova Corso Dogali 1M 16136 Genova Italy; ^98^ Dipartimento di Biologia Università di Napoli Federico II Campus Monte S. Angelo Via Cinthia 4 80126 Napoli Italy; ^99^ Universidade Federal de Pelotas (UFPel) PO Box 354 CEP 96010‐900 Pelotas RS Brazil; ^100^ Astron Environmental Services 129 Royal Street East Perth WA 6004 Australia; ^101^ Department of Environment and Agriculture Curtin University Kent Street Bentley WA 6102 Australia; ^102^ Mount Holyoke College Department of Biological Sciences South Hadley Massachusetts 01075 U.K; ^103^ School of Biological Science University of Plymouth Drake's Circus Plymouth PL4 8AA U.K; ^104^ 351 False Bay Drive Friday Harbor Washington 98250 Malaysia; ^105^ International University of Malaya‐Wales Jalan Tun Ismail 50480 Kuala Lumpur Malaysia; ^106^ Coordenação de Botânica Museu Paraense Emílio Goeldi Caixa Postal 399 CEP 66040‐170 Belém Pará Brazil; ^107^ School of Environmental Sciences University of East Anglia Norwich Research Park Norwich NR4 7TJ U.K; ^108^ Natural Resources Canada Canadian Forest Service Laurentian Forestry Centre 1055 du P.E.P.S. PO Box 10380 Québec QC G1V 4C7 Canada; ^109^ Animal & Environmental Research Group Department of Life Sciences Anglia Ruskin University East Road Cambridge CB1 1PT U.K; ^110^ University of Tromsø Department of Arctic and Marine Biology 9037 Tromsø Norway; ^111^ Universidad Nacional Experimental de Guayana Apdo. Postal 8050 Puerto Ordaz 8015 Estado Bolívar Venezuela; ^112^ Agroscope Reckenholzstr. 191 8046 Zurich Switzerland; ^113^ Corporación Sentido Natural Carrera 70H No. 122 – 98 Apartamento 101 Bogotá Colombia; ^114^ Escuela de Ingeniería Forestal Tecnológico de Costa Rica Apartado 159‐7050 Cartago Costa Rica; ^115^ Asociación para la Conservación y el Estudio de la Biodiversidad (ACEBIO) Casa 15, Barrio Los Abogados Zapote San José Costa Rica; ^116^ International Rice Research Institute DAPO Box 7777 Metro Manila The Philippines; ^117^ University of Debrecen Department of Ecology PO Box 71 4010 Debrecen Hungary; ^118^ Department of Ecology Environment and Plant Sciences Stockholm University 106 91 Stockholm Sweden; ^119^ Instituto de Investigaciones y Recursos Biológicos Alexander von Humboldt Bogotá Colombia; ^120^ Hiroshima University Graduate School of Education 1‐1‐1, Kagamiyama Higashi‐Hiroshima 739‐8524 Japan; ^121^ Scarab Research Group University of Pretoria Pretoria South Africa; ^122^ Centro de Investigaciones en Ecosistemas Universidad Nacional Autónoma de México A.P. 27‐3 Santa María de Guido Morelia Michoacán, México C.P. 58090 Mexico; ^123^ Department of Animal Ecology Justus‐Liebig University Heinrich‐Buff‐Ring 26‐32 35392 Giessen Germany; ^124^ Swedish University of Agricultural Sciences Department of Ecology Box 7044 750 07 Uppsala Sweden; ^125^ Yukon Department of Environment P.O. Box 2703 Whitehorse YT Y1A 2C6 Canada; ^126^ Nature Conservation Foundation Mysore India; ^127^ Department of Environmental & Natural Resources Management University of Patras Seferi 2 30100 Agrinio Greece; ^128^ Centre for Tropical Environmental and Sustainability Science (TESS) and School of Marine and Tropical Biology James Cook University Cairns Qld Australia; ^129^ School of Science and Technology Pacific Adventist University Port Moresby Papua New Guinea; ^130^ Institute of Systematic Botany University of Zurich Zollikerstrasse 107 8008 Zurich Switzerland; ^131^ Institute of Ecology and Evolution University of Bern Baltzerstrasse 6 3012 Bern Switzerland; ^132^ Institute of Ecology University of Bremen FB2, Leobener Str. 28359 Bremen Germany; ^133^ MTA‐ELTE‐MTM Ecology Research Group Pázmány Péter s. 1/c Budapest 1117 Hungary Germany; ^134^ Field Station Fabrikschleichach, Biocenter University of Würzburg Glasshüttenstr. 5 96181 Rauhenebrach Germany; ^135^ Swiss Federal Institute for Forest Snow and Landscape Research WSL Zürcherstrasse 11 8903 Birmensdorf Switzerland; ^136^ Instituto Nacional de Tecnología Agropecuaria EEA Bariloche 8400 Bariloche Argentina; ^137^ INRA, UR 406 Abeilles et Environnement F‐84914 Avignon France; ^138^ Department of Biology San Francisco State University 1600 Holloway Ave San Francisco California 94132; ^139^ Laboratoire de diagnostic en phytoprotection Ministère de l'agriculture, des pêcheries et de l'alimentation du Québec 2700 rue Einstein QC G1P 3W8 Canada; ^140^ Purchase College (State University of New York) 735 Anderson Hill Road Purchase New York 10577 U.K; ^141^ The James Hutton Institute Craigiebuckler Aberdeen, AB15 8QH U.K; ^142^ Universidad de Antioquia Calle 67 No. 53 – 108 Medellín Colombia; ^143^ International Center for Agricultural Research in the Dry Areas (ICARDA) P.O. Box 950764 Amman 11195 Jordan; ^144^ Aarhus University Department of Agroecology Flakkebjerg Research Centre Forsøgsvej 1 4200 Slagelse Denmark; ^145^ Castilla La Mancha University School of Advanced Agricultural Engineering Department of Agroforestry Technology and Science and Genetics Campus Universitario s/n C.P. 02071 Albacete Spain; ^146^ Universidad Autónoma de Nayarit Unidad Académica de Turismo Coordinación de Investigación y Posgrado Ciudad de la Cultura Amado Nervo s/n C.P. 63155 Tepic Nayarit Mexico; ^147^ Graduate School of Agricultural Science Kobe University Kobe 657‐8501 Japan; ^148^ Hortobágy National Park Directorate, 4002 Debrecen P.O.Box 216 Hungary; ^149^ Fauna & Flora International Philippines #8 Foggy Heights Subdivision San Jose Tagaytay City 4120 Philippines; ^150^ De La Salle University‐Dasmariñas West Ave Dasmariñas 4115 Philippines; ^151^ Department of Geography University of Wisconsin‐Madison 550 North Park Street Madison Wisconsin 53706 U.K; ^152^ Marshall Agroecology Ltd, 2 Nut Tree Cottages Barton Winscombe, BS25 1DU U.K; ^153^ Escuela de Posgrados Facultad de Agronomía Doctorado en Agroecología, Universidad Nacional de Colombia, Cra 30 No. 45‐03, Ciudad Universitaria Bogotá Colombia; ^154^ The University of Queensland School of Biological Sciences Brisbane Qld 4120 Australia; ^155^ Swedish University of Agricultural Sciences Department of Ecology Grimsö Wildlife Research Station 730 91 Riddarhyttan Sweden; ^156^ Rainforest Alliance 233 Broadway 28th Floor New York City New York 10279 Kenya; ^157^ Department of Natural Resources and Environmental Sciences N‐407 Turner Hall, MC‐047 1102 South Goodwin Ave. Urbana Illinois 61801 Kenya; ^158^ National Museums of Kenya Botany Department P.O. Box 40658 00100 Nairobi Kenya; ^159^ Department of Zoology National Museums of Kenya P.O. Box 40658 00100 Nairobi Kenya; ^160^ WWF 1250 24th Street NW Washington District of Columbia 20037 China; ^161^ Key Laboratory of Tropical Forest Ecology Xishuangbanna Tropical Botanical Garden CAS, Menglun Mengla Yunnan 666303 China; ^162^ Forestry and Forest Products Research Institute Matsunosato 1 Tsukuba Ibaraki 305–8687 Japan; ^163^ Laboratorio de Investigaciones en Abejas Departamento de Biología Facultad de Ciencias Universidad Nacional de Colombia Sede Bogotá Colombia; ^164^ El Colegio de la Frontera Sur Carretera Panamericana y Periférico Sur S/N. 29290 Chiapas Mexico; ^165^ Department of Biosciences and Department of Environmental Sciences Urban Ecology Research Group University of Helsinki Viikinkaari 2a P.O. Box 65 FI‐00014 Helsinki Finland; ^166^ School of Biology The University of Nottingham University Park Nottingham NG7 2RD U.K; ^167^ Laboratorio de Zoología y Ecología Acuática – LAZOEA Universidad de Los Andes Bogotá Colombia; ^168^ BIO‐Diverse Ließemer Str. 32 a 53179 Bonn Germany; ^169^ Oxford University Centre for the Environment University of Oxford South Parks Road, Oxford OX1 3QY U.K; ^170^ Department of Wildlife and Range Management Kwame Nkrumah University of Science and Technology Kumasi Ghana; ^171^ Forestry Research Institute of Ghana Kumasi Ghana; ^172^ Department of Animal & Environmental Biology University of Benin Benin City Nigeria; ^173^ The Royal Society for the Protection of Birds (RSPB) The Lodge Sandy Bedfordshire, SG19 2DL U.K; ^174^ Laboratorio Ecotono, CONICET–INIBIOMA Universidad Nacional del Comahue Quintral 1250 Bariloche 8400 Argentina; ^175^ Departamento de Biologia Faculdade de Filosofia Ciências e Letras de Ribeirão Preto Universidade de São Paulo Avenida. Bandeirantes 3900 – CEP 14040‐901 – Bairro Monte Alegre Ribeirão Preto SP Brazil; ^176^ Laboratorio de Investigaciones en Abejas‐LABUN Departamento de Biología Facultad de Ciencias Universidad Nacional de Colombia Carrera 45 N° 26‐85, Edificio Uriel Gutiérrez Bogotá DC Colombia Argentina; ^177^ Instituto de Diversidad y Ecología Animal (CONICET‐UNC) and Centro de Zoología Aplicada (UNC) Rondeau 798 X5000AVP Córdoba Argentina; ^178^ School of Environmental Sciences University of East Anglia Norwich NR4 7TJ U.K; ^179^ Lund University Department of Biology/Biodiversity Ecology Building 223 62 Lund Sweden; ^180^ Laboratory of Biogeography & Ecology Department of Geography University of the Aegean 81100 Mytilene Greece; ^181^ Department of Zoology University of Cambridge Cambridge CB2 3EJ U.K; ^182^ Department of Biology Western Kentucky University 1906 College Heights Blvd. Bowling Green Kentucky 42101 Ireland; ^183^ Entomology Cornell University 4126 Comstock Hall Ithaca New York 14850 Ireland; ^184^ School of Natural Sciences Trinity College Dublin College Green Dublin 2 Ireland; ^185^ Center for Environmental Sciences and Engineering & Department of Ecology and Evolutionary Biology University of Connecticut 3107 Horsebarn Hill Road Storrs Connecticut 06269‐4210 Portugal; ^186^ IN+, Instituto Superior Técnico Universidade de Lisboa Av. Rovisco Pais 1 1049‐001 Lisboa Portugal; ^187^ CRA‐ABP, Consiglio per la Ricerca e la sperimentazione in Agricoltura Centro di ricerca per l'agrobiologia e la pedologia Via Lanciola 12/A 50125 – Cascine del Riccio Firenze Italy; ^188^ The Royal Society for the Protection of Birds (RSPB) 2 Lochside View, Edinburgh Park Edinburgh EH12 9DH U.K; ^189^ Department of Forest Ecosystems and Society Oregon State University Corvallis Oregon 97331 Brazil; ^190^ Universidade Federal de Sergipe Cidade Universitária Prof. José Aloísio de Campos Jardim Rosa Elze São Cristóvão Brazil; ^191^ Centro de Ciências Biológicas e da Saúde Universidade Federal de Mato Grosso do Sul P.O Box 549 79070‐900 Campo Grande Brazil; ^192^ 165 Braid Road Edinburgh EH10 6JE U.K; ^193^ Associate Scientist Luquillo LTER Institute for Tropical Ecosystem Studies College of Natural Sciences University of Puerto Rico at Rio Piedras P.O. Box 70377 San Juan Puerto Rico 00936‐8377 Argentina; ^194^ PROPLAME‐PRHIDEB‐CONICET, Departamento de Biodiversidad y Biología Experimental Facultad de Ciencias Exactas y Naturales Universidad de Buenos Aires Ciudad Universitaria PB II, 4to piso (CP1428EHA) Ciudad Autónoma de Buenos Aires Argentina; ^195^ ECT Oekotoxikologie GmbH Böttgerstr. 2‐14 65439 Flörsheim Germany; ^196^ Universidad de Ciencias Aplicadas y Ambientales U.D.C.A. Cl 222 No. 55‐37 Bogotá Colombia; ^197^ School of Biological and Chemical Sciences Queen Mary University of London Mile End Road London E3 5GN U.K; ^198^ Department of Environmental Sciences University of Virginia Charlottesville Virginia 22904‐4123 Canada; ^199^ Blandy Experimental Farm 400 Blandy Farm Lane Boyce Virginia 22620 Canada; ^200^ Département des sciences biologiques Université du Québec à Montréal (UQAM) Case postale 8888, Succursale Centre‐ville Montréal QC H3C 3P8 Canada; ^201^ School of Geography Earth and Environmental Sciences University of Birmingham Birmingham B15 2TT U.K; ^202^ Institute of Silviculture and Forest Protection University of West Hungary Bajcsy‐Zsilinszky u. 4. 9400 Sopron, Hungary; ^203^ Red de Ecología Funcional Instituto de Ecología A.C. Carretera Antigua a Coatepec N° 351 El Haya, CP 91070 Xalapa, Veracruz Mexico; ^204^ Stockholm University Department of Ecology Environment and Plant Sciences SE 106 91 Stockholm Sweden; ^205^ Helmholtz Centre for Environmental Research – UFZ Theodor‐Lieser‐Strasse 4 06120 Halle Germany; ^206^ Lawrence University 711 E. Boldt Way Appleton Wisconsin 54911 India; ^207^ School of Human Ecology Dr. B.R. Ambedkar University Lothian Road Delhi 110006 India; ^208^ Department of Ecology and Natural Resource Management (INA) Norwegian University of Life Sciences (NMBU) Box 5003 1432 Ås Norway; ^209^ Center for International Forestry Research Bogor 16000 Indonesia; ^210^ Universidade Federal do Pará Instituto de Ciências Biológicas Rua Augusto Correa, 01 Belém 66075‐110 Pará Brazil; ^211^ Department of Zoology University of Oxford South Parks Road Oxford OX1 3PS U.K; ^212^ USDA – APHIS – PPQ 389 Oyster Point Blvd. Suite 2 South San Francisco California 94080 Colombia Republic of Singapore; ^213^ Universidad Nacional de Colombia Cra. 64 X Cll. 65. Bloque 11, Oficina 207 Medellin Colombia Republic of Singapore; ^214^ Department of Biological Sciences National University of Singapore 14 Science Drive 4 Singapore City 117543 Republic of Singapore; ^215^ EComAS (Grupo de Investigación en Ecología de Comunidades Áridas y Semiáridas) Dpto. de Recursos Naturales Facultad de Ciencias Exactas y Naturales Universidad Nacional de La Pampa Santa Rosa Argentina; ^216^ School of Natural Sciences and Trinity Centre for Biodiversity Research Trinity College Dublin College Green Dublin 2 Ireland; ^217^ Kadoorie Conservation China Kadoorie Farm and Botanic Garden Lam Kam Road, Tai Po New Territories Hong Kong SAR China; ^218^ Department of Resource Management and Geography The University of Melbourne 500 Yarra Boulevard Richmond VIC 3121 Australia; ^219^ Northwestern University Program in Plant Biology and Conservation 2205 Tech Drive O.T. Hogan Hall Room 2‐144 Evanston Illinois 60208 Hungary; ^220^ Chicago Botanic Garden 1000 Lake Cook Road Glencoe Illinois 60022 Hungary; ^221^ MTA‐DE Biodiversity and Ecosystem Services Research Group Egyetem ter 1 Debrecen 4032 Hungary; ^222^ University Museum of Zoology Downing Street Cambridge CB2 3EJ U.K; ^223^ University of Canterbury Private bag 4800 Christchurch 8140 New Zealand; ^224^ NERC Centre for Ecology & Hydrology Bush Estate Penicuik Edinburgh EH26 0QB U.K; ^225^ Institute of Biodiversity and Ecosystem Research Bulgarian Academy of Science 23 Akademik Georgi Bonchev str. Block 23 1113 Sofia Bulgaria; ^226^ Department of Earth and Environmental Science Division Forest, Nature and Landscape KU Leuven Celestijnenlaan 200E 3001 Leuven Belgium; ^227^ Departamento de Ciencias Químico‐Biológicas Universidad de las Américas Puebla 72810 Cholula Puebla Mexico; ^228^ Universidad de Santiago de Chile Avenida Alameda Libertador Bernardo O'Higgins 3363 Estación Central Santiago Chile; ^229^ Spotvogellaan 68 2566 PN The Hague The Netherlands; ^230^ Dillon Consulting Limited 137 Chain Lake Drive Halifax NS B3S 1B3 Canada; ^231^ The Key Laboratory of Conservation Biology for Endangered Wildlife of the Ministry of Education College of Life Sciences Zhejiang University Hangzhou 310058 China; ^232^ University of Florida 3205 College Avenue Fort Lauderdale Florida 33314 Australia; ^233^ The Environment Institute and School of Earth and Environmental Sciences The University of Adelaide SA 5005 Australia; ^234^ Institute of Experimental Ecology University of Ulm Albert‐Einstein‐Allee 11 89069 Ulm Germany; ^235^ Behavioural Ecology and Biocontrol Department of Biology National University of Ireland Maynooth Co. Kildare Ireland; ^236^ Center for Environmental Sciences & Engineering University of Connecticut 3107 Horsebarn Hill Road Storrs Connecticut 06269‐4210 Australia; ^237^ Department of Ecology & Evolutionary Biology University of Connecticut 3107 Horsebarn Hill Road Storrs Connecticut 06269‐4210 Australia; ^238^ Charles Darwin University 7 Ellengowan Dr Brinkin NT 0810 Australia; ^239^ University of Amsterdam Institute for Biodiversity and Ecosystem Dynamics (IBED) P.O. Box 94248 1090 GE Amsterdam The Netherlands; ^240^ NERC Centre for Ecology & Hydrology Crowmarsh Gifford Wallingford Oxfordshire OX10 8BB U.K; ^241^ University of East Anglia Norwich Research Park Norwich Norfolk, NR4 7TJ U.K; ^242^ Kunming Institute of Zoology Kunming Yunnan, 650023 China; ^243^ Institute of Animal Ecology Justus‐Liebig‐University Heinrich‐Buff‐Ring 26 35392 Giessen Germany; ^244^ A. N. Severtsov Institute of Ecology and Evolution Leninsky Prospekt 33 119071 Moscow Russia; ^245^ Centre for Biodiversity and Environment Research Department of Genetics Evolution and Environment University College London Gower Street London WC1E 6BT U.K

**Keywords:** Data sharing, global change, habitat destruction, land use

## Abstract

Biodiversity continues to decline in the face of increasing anthropogenic pressures such as habitat destruction, exploitation, pollution and introduction of alien species. Existing global databases of species’ threat status or population time series are dominated by charismatic species. The collation of datasets with broad taxonomic and biogeographic extents, and that support computation of a range of biodiversity indicators, is necessary to enable better understanding of historical declines and to project – and avert – future declines. We describe and assess a new database of more than 1.6 million samples from 78 countries representing over 28,000 species, collated from existing spatial comparisons of local‐scale biodiversity exposed to different intensities and types of anthropogenic pressures, from terrestrial sites around the world. The database contains measurements taken in 208 (of 814) ecoregions, 13 (of 14) biomes, 25 (of 35) biodiversity hotspots and 16 (of 17) megadiverse countries. The database contains more than 1% of the total number of all species described, and more than 1% of the described species within many taxonomic groups – including flowering plants, gymnosperms, birds, mammals, reptiles, amphibians, beetles, lepidopterans and hymenopterans. The dataset, which is still being added to, is therefore already considerably larger and more representative than those used by previous quantitative models of biodiversity trends and responses. The database is being assembled as part of the PREDICTS project (Projecting Responses of Ecological Diversity In Changing Terrestrial Systems – www.predicts.org.uk). We make site‐level summary data available alongside this article. The full database will be publicly available in 2015.

## Graphical abstract

The collation of biodiversity datasets with broad taxonomic and biogeographic extents is necessary to understand historical declines and to project – and hopefully avert – future declines. We describe a newly collated database of more than 1.6 million biodiversity measurements from 78 countries representing over 28,000 species, collated from existing spatial comparisons of local‐scale biodiversity exposed to different intensities and types of anthropogenic pressures, from terrestrial sites around the world.

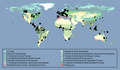

## Introduction

Despite the commitment made by the Parties to the Convention on Biological Diversity (CBD) to reduce the rate of biodiversity loss by 2010, global biodiversity indicators show continued decline at steady or accelerating rates, while the pressures behind the decline are steady or intensifying (Butchart et al. 2010; Mace et al. 2010). Evaluations of progress toward the CBD's 2010 target highlighted the need for datasets with broader taxonomic and geographic coverage than existing ones (Walpole et al. 2009; Jones et al. 2011). Taxonomic breadth is needed because species’ ability to tolerate human impacts – destruction, degradation and fragmentation of habitats, the reduction of individual survival and fecundity through exploitation, pollution and introduction of alien species – varies among major taxonomic groups (Vié et al. 2009). For instance, the proportion of species listed as threatened in the IUCN Red List is much higher in amphibians than in birds (International Union for Conservation of Nature 2013). Geographic breadth is needed because human impacts show strong spatial variation: most of Western Europe has long been dominated by human land use, for example, whereas much of the Amazon basin is still close to a natural state (Ellis et al. 2010). Thus, in the absence of broad coverage, any pattern seen in a dataset is prone to reflect the choice of taxa and region as much as true global patterns and trends.

The most direct way to capture the effects of human activities on biodiversity is by analysis of time‐series data from ecological communities, assemblages or populations, relating changes in biodiversity to changes in human activity (Vačkář 2012). However, long‐term data suitable for such modeling have limited geographic and taxonomic coverage, and often record only the presence or absence of species (e.g., Dornelas et al. 2013). Time‐series data are also seldom linked to site‐level information on drivers of change, making it hard to use such data to model biodiversity responses or to project responses into the future. Ecologists have therefore more often analyzed spatial comparisons among sites that differ in the human impacts they face. Although the underlying assumption that biotic differences among sites are caused by human impacts has been criticized (e.g., Johnson and Miyanishi 2008; Pfeifer et al. 2014), it is more likely to be reasonable when the sites being compared are surveyed in the same way, when they are well matched in terms of other potentially important variables (e.g., Blois et al. 2013; Pfeifer et al. 2014), when analyses focus on community‐level summaries rather than individual species (e.g., Algar et al. 2009), and when the spatial and temporal variations being considered are similar in magnitude (Blois et al. 2013). Collations of well‐matched site surveys therefore offer the possibility of analyzing how biodiversity is responding to human impacts without losing taxonomic and geographic breadth.

Openness of data is a further important consideration. The reproducibility and transparency that open data can confer offer benefits to all areas of scientific research, and are particularly important to research that is potentially relevant to policy (Reichman et al. 2011). Transparency has already been highlighted as crucial to the credibility of biodiversity indicators and models (e.g., UNEP‐WCMC 2009; Feld et al. 2010; Heink and Kowarik 2010) but the datasets underpinning previous policy‐relevant analyses have not always been made publicly available.

We present a new database that collates published, in‐press and other quality‐assured spatial comparisons of community composition and site‐level biodiversity from terrestrial sites around the world. The underlying data are made up of abundance, presence/absence and species‐richness measures of a wide range of taxa that face many different anthropogenic pressures. As of March 2014, the dataset contains more than 1.6 million samples from 78 countries representing over 28,000 species. The dataset, which is still being added to, is being assembled as part of the PREDICTS project (Projecting Responses of Ecological Diversity In Changing Terrestrial Systems – http://www.predicts.org.uk), the primary purpose of which is to model and project how biodiversity in terrestrial communities responds to human activity. The dataset is already considerably larger and more representative than those used in existing quantitative models of biodiversity trends such as the Living Planet Index (WWF International 2012) and GLOBIO3 (Alkemade et al. 2009).

In this paper we introduce the database, describe in detail how it was collated, validated and curated, and assess its taxonomic, geographic and temporal coverage. We make available a summary dataset that contains, for each sampling location, the predominant land use, land‐use intensity, type of habitat fragmentation, geographic coordinates, sampling dates, country, biogeographic realm, ecoregion, biome, biodiversity hotspot, taxonomic group studied and the number of measurements taken. The full dataset constitutes a large evidence base for the analysis of:

- The responses of biodiversity to human impacts for different countries, biomes and major taxonomic groups;
- The differing responses within and outside protected areas;
- How traits such as body size, range size and ecological specialism mediate responses and
- How human impacts alter community composition.

The summary dataset permits analysis of geographic and taxonomic variation in study size and design. The complete database, which will be made freely available at the end of the current phase of the project in 2015, will be of use to all researchers interested in producing models of how biodiversity responds to human pressures.

## Methods

### Criteria for inclusion

We considered only data that met all of the following criteria:

- Data are published, in press or were collected using a published methodology;
- The paper or report presents data about the effect of one or more human activities on one or more named taxa, and where the degree of human activity differed among sampling locations and/or times;
- Some measure of overall biodiversity, or of the abundance or occurrence of the named taxa, was made at two or more sampling locations and/or times;
- Measurements within each data source were taken using the same sampling procedure, possibly with variation in sampling effort, at each site and time;
- The paper reported, or authors subsequently provided, geographical coordinates for the sites sampled.

One of the modeling approaches used by PREDICTS is to relate diversity measurements to remotely sensed data, specifically those gathered by NASA's Moderate Resolution Imaging Spectroradiometer (MODIS) instruments (Justice et al. 1998). MODIS data are available from early 2000 onwards so, after a short initial data collation stage, we additionally required that diversity sampling had been completed after the beginning of 2000.

Where possible, we also obtained the following (see Site characteristics, below, for more details):

- The identities of the taxa sampled, ideally resolved to species level;
- The date(s) on which each measurement was taken;
- The area of the habitat patch that encompassed each site;
- The maximum linear extent sampled at the site;
- An indication of the land use at each site, e.g. primary, secondary, cropland, pasture;
- Indications of how intensively each site was used by people;
- Descriptions of any transects used in sampling (start point, end point, direction, etc.);
- Other information about each site that might be relevant to modeling responses of biodiversity to human activity, such as any pressures known to be acting on the site, descriptions of agriculture taking place and, for spatially blocked designs, which block each site was in.

### Searches

We collated data by running sub‐projects that investigated different regions, taxonomic groups or overlapping anthropogenic pressures: some focused on particular taxa (e.g., bees), threatening processes (e.g., habitat fragmentation, urbanization), land‐cover classes (e.g., comparing primary, secondary and plantation tropical forests), or regions (e.g., Colombia). We introduced the project and requested data at conferences and in journals (Newbold et al. 2012; Hudson et al. 2013). After the first six months of broad searching, we increasingly targeted efforts toward under‐represented taxa, habitat types, biomes and regions. In addition to articles written in English, we also considered those written in Mandarin, Spanish and Portuguese – languages in which one or more of our data compilers were proficient.

### Data collection

To maximize consistency in how incoming data were treated, we developed customized metadata and data capture tools – a PDF form and a structured Excel file – together with detailed definitions and instructions on their usage. The PDF form was used to capture bibliographic information, corresponding author contact details and meta‐data such as the country or countries in which data were collected, the number of taxa sampled, the number of sampling locations and the approximate geographical center(s) of the study area(s). The Excel file was used to capture details of each sampling site and the diversity measurements themselves. The PDF form and Excel file are available in Supplementary Information. We wrote software that comprehensively validates pairs of PDF and Excel files for consistency; details are in the “Database” section.

Most papers that we considered did not publish all the information that we required; in particular, site coordinates and species names were frequently not published. We contacted authors for these data and to request permission to include their contributed data in the PREDICTS database. We used the insightly customer relationship management application (https://www.insightly.com/) to manage contact with authors.

### Structure of data

We structured data into Data Sources, Studies, and Sites. The highest level of organization is the Data Source. A Data Source typically represents data from a single published paper, although in some cases the data were taken from more than one paper, from a non‐governmental organization report or from a PhD or MSc thesis. A Data Source contains one or more Studies. A Study contains two or more Sites, a list of taxa that were sampled and a site‐by‐species matrix of observations (e.g., presence/absence or abundance). All diversity measurements within a Study must have been collected using the same sampling method. For example, a paper might present, for the same set of Sites, data from pitfall traps and from Malaise traps. We would structure these data into a single Data Source containing two Studies – one for each trapping technique. It is therefore reasonable to directly compare observations within a Study but not, because of methodological differences, among Studies. Sometimes, the data presented in a paper were aggregates of data from multiple sampling methods. In these cases, provided that the same set of sampling methods was applied at each Site, we placed the data in a single Study.

We classified the diversity observations as abundance, occurrence or species richness. Some of the site‐by‐species matrices that we received contained empty cells, which we interpreted as follows: (1) where the filled‐in values in the matrix were all non‐zero, we interpreted blanks as zeros or (2) where some of the values in the matrix were zero, we took empty cells as an indication that the taxa concerned were not looked for at those Sites, and interpreted empty cells as missing values.

Where possible, we recorded the sampling effort expended at each Site and allowed the units of sampling effort to vary among Studies. For example, if transects had been used, the (Study‐level) sampling effort units might be meters or kilometers and the (Site‐level) sampling efforts might be the length of the transects. If pitfall traps had been used, the (Study‐level) sampling effort units might be “number of trap nights” and the (Site‐level) sampling efforts might be the number of traps used multiplied by the number of nights that sampling took place. Where possible, we also recorded an estimate of the maximum linear extent encompassed by the sampling at each Site – the distance covered by a transect, the distance between two pitfall traps or the greatest linear extent of a more complex sampling design (see Figure S1 in Supplementary Information for details).

### Site characteristics

We recorded each Site's coordinates as latitude and longitude (WGS84 datum), converting where necessary from local grid‐based coordinate systems. Where precise coordinates for Sites were not available, we georeferenced them from maps or schemes available from the published sources or provided by authors. We converted each map to a semi‐transparent image that was georeferenced using either ArcGIS (Environmental Systems Research Institute (ESRI) 2011) or Google Earth (http://www.google.co.uk/intl/en_uk/earth/ ), by positioning and resizing the image on the top of ArcGIS Online World Imagery or Google Maps until we achieved the best possible match of mapped geographical features with the base map. We then obtained geographic coordinates using geographic information systems (GIS) for each Site center or point location. We also recorded authors’ descriptions of the habitat at each Site and of any transects walked.

For each Site we recorded the dates during which sampling took place. Not all authors presented precise sampling dates – some gave them to the nearest month or year. We therefore recorded the earliest possible start date, the latest possible end date and the resolution of the dates that were given to us. Where dates were given to the nearest month or year, we recorded the start and end dates as the earliest and latest possible day, respectively. For example, if the authors reported that sampling took place between June and August of 2007, we recorded the date resolution as “month,” the start of sampling as June 1, 2007 and end of sampling as August 31, 2007. This scheme meant that we could store sampling dates using regular database structures (which require that the year, month, and day are all present), while retaining information about the precision of sampling dates that were given to us.

We assigned classifications of predominant land use and land‐use intensity to each Site. Because of PREDICTS’ aim of making projections about the future of biodiversity under alternative scenarios, our land‐use classification was based on five classes defined in the Representative Concentration Pathways harmonized land‐use estimates (Hurtt et al. 2011) – primary vegetation, secondary vegetation, cropland, pasture and urban – with the addition of plantation forest to account for the likely differences in the biodiversity of natural forest and plantation forest (e.g., Gibson et al. 2011) and a “Cannot decide” category for when insufficient information was available. Previous work has suggested that both the biodiversity and community composition differ strongly between sites in secondary vegetation of different maturity (Barlow et al. 2007); therefore, we subdivided secondary vegetation by stage – young, intermediate, mature and (when information was lacking) indeterminate – by considering vegetation structure (not diversity). We used authors’ descriptions of Sites, when provided, to classify land‐use intensity as minimal, light or intense, depending on the land use in question, again with “Cannot decide” as an option for when information was lacking. A detailed description of how classifications are assigned is in the Supplementary section “Notes on assigning predominant land use and use intensity” and Tables S1 and S2.

Given the likely importance of these classifications as explanatory variables in modeling responses of biodiversity to human impacts, we conducted a blind repeatability study in which one person (the last author, who had not originally scored any Sites) rescored both predominant land use and use intensity for 100 Sites chosen at random. Exact matches of predominant land use were achieved for 71 Sites; 15 of the remaining 29 were “near misses” specified in advance (i.e., primary vegetation versus mature secondary; adjacent stages of secondary vegetation; indeterminate secondary versus any other secondary stage; and cannot decide versus any other class). Cohen's kappa provides a measure of inter‐rate agreement, ranging from 0 (agreement no better than random) to 1 (perfect agreement). For predominant land use, Cohen's kappa = 0.662 (if only exact agreement gets credit) or 0.721 (if near misses are scored as 0.5); values in the range 0.6–0.8 indicate “substantial agreement” (Landis and Koch 1977), indicating that our categories, criteria and training are sufficiently clear for users to score Sites reliably. Moving to use intensity, we found exact agreement for 57 of 100 Sites, with 39 of the remaining 43 being “near misses” (adjacent intensity classes, or cannot decide versus any other class), giving Cohen's kappa values of 0.363 (exact agreement only) or 0.385 (near misses scored as 0.5), representing “fair agreement” (Landis and Koch 1977); agreement is slightly higher among the 71 Sites for which predominant land use was matched (exact agreement in 44 of 71 Sites, kappa = 0.428, indicating “moderate agreement”: Landis and Koch 1977).

Where known, we recorded the number of years since conversion to the present predominant land use. If the Site's previous land use was primary habitat, we recorded the number of years since it was converted to the current land use. If the habitat was converted to secondary forest (clear‐felled forest or abandoned agricultural land), we recorded the number of years since it was converted/clear‐felled/abandoned. Where ranges were reported, we used mid‐range values; if papers reported times as “greater than N years” or “at least N years,” we recorded a value of N × 1.25. Based on previous work (Wilcove et al. 1986; Dickman 1987), we assigned one of five habitat fragmentation classes: (1) well within unfragmented habitat, (2) within unfragmented habitat but at or near its edge, (3) within a remnant patch (perhaps at its edge) that is surrounded by other habitats, (4) representative part of a fragmented landscape and (5) part of the matrix surrounding remnant patches. These are described and illustrated in Table S3 and Figure S2. We also recorded the area of the patch of predominant habitat within which the Site was located, where this information was available. We recorded a value of −1 if the patch area was unknown but large, extending far beyond the sampled Site.

### Database

Completed PDF and Excel files were uploaded to a PostgreSQL 9.1 database (PostgreSQL Global Development Group, http://www.postgresql.org/) with the PostGIS 2.0.1 spatial extension (Refractions Research Inc, http://www.postgis.net/). The database schema is shown in Figure S3.

We wrote software in the Python programming language (http://www.python.org/) to perform comprehensive data validation; files were fully validated before their data were added to the database. Examples of lower level invalid data included missing values for mandatory fields, a negative time since conversion, a latitude given as 1° 61’, a date given as 32nd January, duplicated Site names and duplicated taxon names. Commonly encountered higher level problems included mistakes in coordinates, such as latitude and longitude swapped, decimal latitude and longitude incorrectly assembled from DD/MM/SS components, and direction (north/south, east/west) swapped round. These mistakes typically resulted in coordinates that plotted in countries not matching those given in the metadata and/or out to sea. The former was detected automatically by validation software, which required that the GIS‐matched country for each Site (see “Biogeographical coverage ” below) matched the country name entered in the PDF file for the Study; where a Study spanned several countries, we set the country name to “Multiple countries.” We visually inspected all Site locations on a map and compared them to maps presented in the source article or given to us by the authors, catching coordinates that were mistakenly out to sea and providing a check of accuracy.

Our database linked each Data Source to the relevant record in our Insightly contact management database. This allows us to trace each datum back to the email that granted permission for us to include it in our database.

### Biogeographical coverage

In order to assess the data's geographical and biogeographical coverage, we matched each Site's coordinates to GIS datasets that were loaded into our database:

- Terrestrial Ecoregions of the World (The Nature Conservancy 2009), giving the ecoregion, biome and biogeographic realm;
- World Borders 0.3 (Thematic Mapping 2008), giving the country, United Nations (UN) region and UN subregion;
- Biodiversity Hotspots (Conservation International Foundation 2011).

Global GIS layers appear coarse at local scales and we anticipated that Sites on coasts or on islands could fall slightly outside the relevant polygons. Our software therefore matched Sites to the nearest ecoregion and nearest country polygons, and recorded the distance in meters to that polygon, with a value of zero for Sites that fell within a polygon; we reviewed Sites with non‐zero distances. The software precisely matched Sites to hotspot polygons. The relative coarseness of GIS polygons might result in small errors in our assessments of coverage (i.e., at borders between biomes, ecoregions and countries, and at the edges of hotspots) – we expect that these errors should be small in number and unbiased.

We also estimated the yearly value of total net primary production (TNPP) for biomes and five‐degree latitudinal belts, using 2010 spatial (0.1‐degree resolution) monthly datasets “NPP – Net Primary Productivity 1 month‐Terra/MODIS” compiled and distributed by NASA Earth Observations (http://neo.sci.gsfc.nasa.gov/view.php?datasetId=MOD17A2_M_PSN&year=2010). We used the NPP values (average for each month assimilation measured in grams of carbon per square meter per day) to estimate monthly and annual NPP. We then derived TNPP values by multiplying NPP values by the total terrestrial area for that ecoregion/latitudinal belt. We assessed the representativeness of land use and land‐use intensity combinations by comparing the proportion of Sites in each combination to a corresponding estimate of the proportion of total terrestrial area for 2005, computed using land‐use data from the HYDE historical reconstruction (Hurtt et al. 2011) and intensity data from the Global Land Systems dataset (van Asselen and Verburg 2012).

### Taxonomic names and classification

We wanted to identify taxa in our database as precisely as possible and to place them in higher level groups, which required relating the taxonomic names presented in our datasets to a stable and authoritative resource for nomenclature. We used the Catalogue of Life (http://www.catalogueoflife.org/) for three main reasons. First, it provides broad taxonomic coverage. Second, Catalogue of Life publishes Annual Checklists. Third, Catalogue of Life provides a single accepted taxonomic classification for each species that is represented. Not all databases provide this guarantee; for example, Encyclopedia of Life (http://www.eol.org/) provides zero, one or more taxonomic classifications for each represented species. We therefore matched taxonomic names to the Catalogue of Life 2013 Annual Checklist (Roskov et al. 2013, henceforth COL).

There was large variation in the form of the taxonomic names presented in the source datasets, for example:

- A Latin binomial, with and without authority, year and other information;
- A generic name, possibly with a number to distinguish morphospecies from congenerics in the same Study (e.g., “*Bracon* sp. 1”);
- The name of a higher taxonomic rank such as family, order, class;
- A common name (usually for birds), sometimes not in English;
- A textual description, code, letter or number with no further information except an indication of some aspect of higher taxonomy.

Most names were Latin binomials, generic names or morphospecies names. Few binomials were associated with an authority – even when they were, time constraints mean that it would not have been practical to make use of this information. Many names contained typographical errors.

We represented each taxon by three different names: “Name entered,” “Parsed name,” and “COL query name.” “Name entered” was the name assigned to the taxon in the dataset provided to us by the investigators who collected the data. We used the Global Names Architecture's biodiversity package ( https://github.com/GlobalNamesArchitecture/biodiversity) to parse “Name entered” and extract a putative Latin binomial, which we assigned to both “Parsed name” and “COL query name.” For example, the result of parsing the name “*Ancistrocerus trifasciatus* Müll.” was “*Ancistrocerus trifasciatus*.” The parser treated all names as if they were scientific taxonomic names, so the result of parsing common names was not sensible: e.g. “Black and White Casqued Hornbill” was parsed as “Black and.” We expected that common names would be rare – where they did arise, they were detected and corrected as part of our curation process, which is described below. Other examples of the parser's behavior are shown in Table S4.

We queried COL with each “COL query name” and stored the matching COL ID, taxonomic name, rank and classification (kingdom, phylum, class, order, family, genus, species and infraspecies). We assumed that the original authors gave the most authoritative identification of species. Therefore, when a COL search returned more than one result, and the results were made up of one accepted name together with one or more synonyms and/or ambiguous synonyms and/or common names and/or misapplied names, our software recorded the accepted name. For example, COL returns three results for the salticid spider *Euophrys frontalis* – one accepted name and two synonyms.

When a COL search returned more than one result, and the results included zero or two or more accepted names, we used the lowest level of classification common to all results. For example, COL lists *Notiophilus* as an accepted genus in two beetle families – Carabidae and Erirhinidae. This is a violation of the rules of nomenclature, but taxonomic databases are imperfect and such violations are to be expected. In this case, the lowest rank common to both families is the order Coleoptera.

#### Curating names

We reviewed:

- Taxa that had no matching COL record;
- Taxa that had a result at a rank higher than species and a “Name entered” that was either a Latin binomial or a common name;
- Cases where the same “Parsed name” in different Studies linked to different COL records;
- Studies for which the lowest common taxonomic rank did not seem appropriate; for example, a Study of birds should have a lowest common taxonomic rank of class Aves or lower rank within Aves.

Where a change was required, we altered “COL query name”, recording the reason why the change was made, and reran the COL query. Sometimes, this curation step had to be repeated multiple times. In all cases, we retained the names given to us by the authors, in the “Name entered” and “Parsed name” columns.

Typographical errors were the most common cause for failed COL searches; for example, the hymenopteran *Diphaglossa gayi* was given as *Diphaglosa gayi*. Such errors were detected by visual inspection and by performing manual searches on services that perform fuzzy matching and suggest alternatives, such as Google and Encyclopedia of Life. In cases where “Parsed name” was a binomial without typographical errors but that was not recognized by COL, we searched web sites such as Encyclopedia of Life and The Plant List (http://www.theplantlist.org/) for synonyms and alternative spellings and queried COL with the results. Where there were no synonyms or where COL did not recognize the synonyms, we searched COL for just the genus. If the genus was not recognized by COL, we used the same web services to obtain higher level ranks, until we found a rank that COL recognized.

Some names matched COL records in two different kingdoms. For example, *Bellardia*,* Dracaena* and *Ficus* are all genera of plants and of animals. In such cases, we instructed our software to consider only COL records from the expected kingdom. We also constrained results when a name matched COL records in two different branches within the same kingdom; for example, considering the *Notiophilus* example given above – if the Study was of carabid beetles, we would instruct of software to consider only results within family Carabidae.

COL allows searches for common names. Where “Name entered” was a common name that was not recognized by COL, we searched web sites as described above and set “COL query name” to the appropriate Latin binomial.

Some studies of birds presented additional complications. Some authors presented taxon names as four‐letter codes that are contractions of common names (e.g., AMKE was used by Chapman and Reich (2007) to indicate *Falco sparverius*, American kestrel) or of Latin binomials (e.g., ACBA was used by Shahabuddin and Kumar (2007) to indicate *Accipiter badius*). Some of these codes are valid taxonomic names in their own right. For example, Shahabuddin and Kumar (2007) used the code TEPA to indicate the passerine *Terpsiphone paradisi*. However, *Tepa* is also a genus of Hemiptera. Left uncurated, COL recognized TEPA as the hemipteran genus and the Study consequently had a lowest common taxonomic rank of kingdom Animalia, not of class Aves or a lower rank within Aves, as we would expect. Some codes did not appear on published lists (e.g., http://www.birdpop.org/alphacodes.htm, http://www.pwrc.usgs.gov/bbl/manual/speclist.cfm, http://www.carolinabirdclub.org/bandcodes.html and http://infohost.nmt.edu/~shipman/z/nom/bbs.html) or in the files provided by the authors, either because of typographical errors, omissions or incomplete coverage. Fortunately, codes are constructed by following a simple set of rules – the first two letters of the genus and species of binomials, and a slightly more complex method for common names of North American birds (http://infohost.nmt.edu/~shipman/z/nom/bblrules.html). We cautiously reverse‐engineered unrecognized codes by following the appropriate rules and then searched lists of birds of the country concerned for possible matches. For example, we deduced from the Wikipedia list of birds of India (http://en.wikipedia.org/wiki/List_of_birds_of_India) that KEZE – used in a study of birds in Rajasthan, northwestern India (Shahabuddin and Kumar 2007) – most likely indicates *Ketupa zeylonensis*. Another problem is that collisions occur – the same code can apply to more than one taxon. For example, PEPT is the accepted code for *Atalotriccus pilaris* (pale‐eyed pygmy tyrant – http://www.birdpop.org/alphacodes.htm), a species that occurs in the Neotropics. The same code was used by the Indian study of Shahabuddin and Kumar (2007) to indicate *Pernis ptilorhynchus* (crested honey buzzard). We therefore reverse‐engineered bird codes on a case‐by‐case basis. Where a code could represent more than one species, we set “COL query name” as the lowest taxonomic rank common to all matching species.

#### Counting the number of species

It was not possible to precisely count the number of species represented in our database because of ambiguity inherent in the taxon names provided with the data. We estimated the number of species as follows. Names with a COL result at either species or infraspecies level were counted once per name. Names with a COL result resolved to higher taxonomic ranks were counted once per Study. To illustrate this scheme, consider the bat genus *Eonycteris*, which contains three species. Suppose that Study A sampled all three species and that the investigators could distinguish individuals as belonging to three separate species but could not assign them to named species, reporting them as *Eonycteris* sp. 1, *Eonycteris* sp. 2 and *Eonycteris* sp. 3. Study B also sampled all three species of *Eonycteris* and again reported *Eonycteris* sp. 1, *Eonycteris* sp. 2 and *Eonycteris* sp. 3. We would erroneously consider these taxa to be six different species. We did not attempt to determine how often, if at all, such inflation occurred.

In order to assess the taxonomic coverage of our data, we computed a higher taxonomic grouping for each taxon as: (1) order where class was Insecta or Entognatha; (2) class where phylum was Arthropoda (excluding Insecta), Chordata or Tracheophyta; otherwise 3) phylum. So the higher taxonomic group of a bee is order Hymenoptera (following rule 1), the higher taxonomic group of a wolf is class Mammalia (rule 2), and the higher taxonomic group of a snail is phylum Gastropoda (rule 3). For each higher taxonomic group, we compared the numbers of species in our database to the estimated number of described species presented by Chapman (2009). Some of the higher taxonomic groups that we computed did not directly relate to the groups presented by Chapman (2009) so, in order to compare counts, we computed Magnoliophyta as the sum of Magnoliopsida and Liliopsida; Gymnosperms as the sum of Pinopsida and Gnetopsida; Ferns and allies as the sum of Polypodiopsida, Lycopodiopsida, Psilotopsida, Equisetopsida and Marattiopsida; and Crustacea as Malacostraca.

For some of our analyses, we related taxonomic names to databases of species’ traits. To do this, we synthesized, for each taxon, a “Best guess binomial”:

- The COL taxon name if the COL rank was Species;
- The first two words of the COL taxon if the rank was Infraspecies;
- The first two words of “Parsed name” if the rank was neither Species nor Infraspecies and “Parsed name” contained two or more words;
- Empty in other cases.

This scheme meant that even though COL did not recognize all of the Latin binomials that were given to us, we could maximize matches between names in our databases with names in the species’ trait databases.

## Results

**Figure 1:**
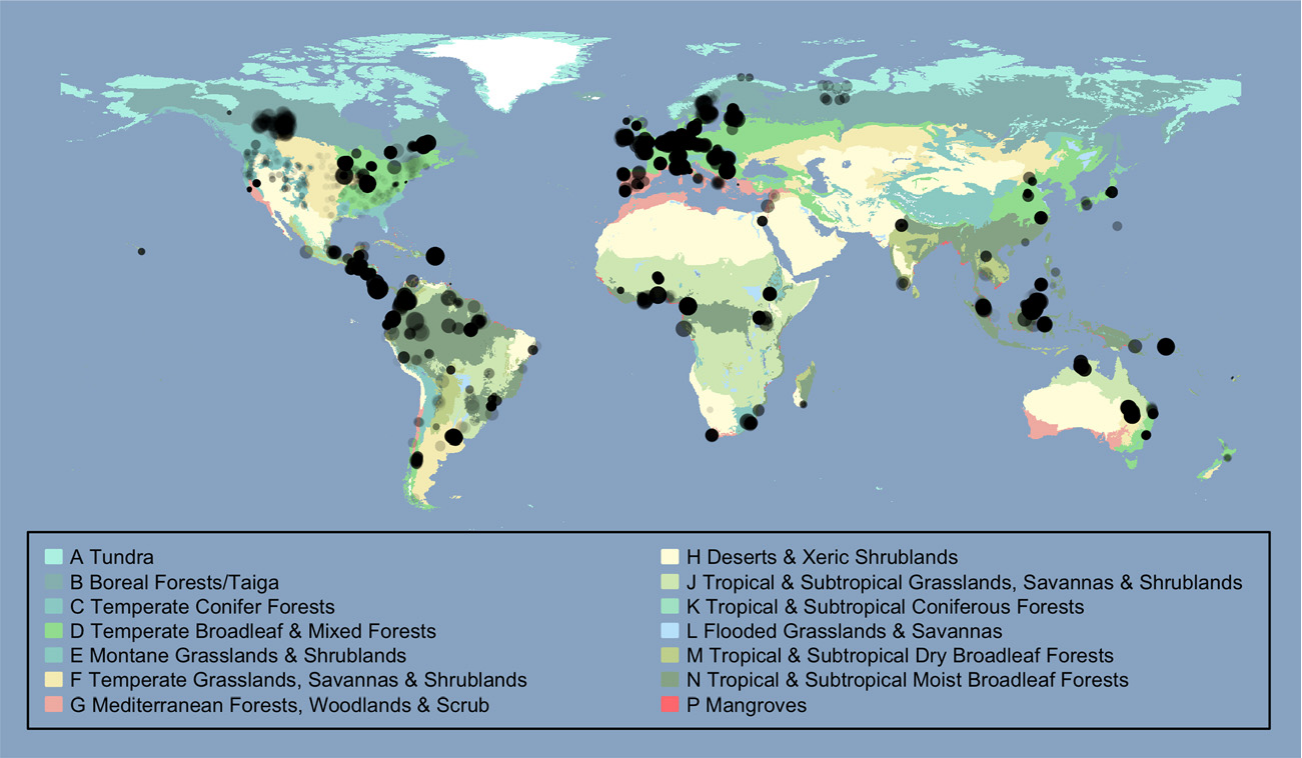
Site locations. Colors indicate biomes, taken from The Nature Conservancy's (2009) terrestrial ecoregions of the world dataset, shown in a geographic (WGS84) projection. Circle radii are proportional to log_10_ of the number of samples at that Site. All circles have the same degree of partial transparency.

Between March 2012 and March 2014, we collated data from 284 Data Sources, 407 Studies and 13,337 Sites in 78 countries and 208 (of 814) ecoregions (Fig. 1). The best‐represented UN‐defined subregions are North America (17.51% of Sites), Western Europe (14.14%) and South America (13.37%). As of March 31, 2014, the database contained 1,624,685 biodiversity samples – 1,307,947 of abundance, 316,580 of occurrence and 158 of species richness. The subregions with the most samples are Southeast Asia (24.66%), Western Europe (11.36%) and North America (10.88%).

**Figure 2:**
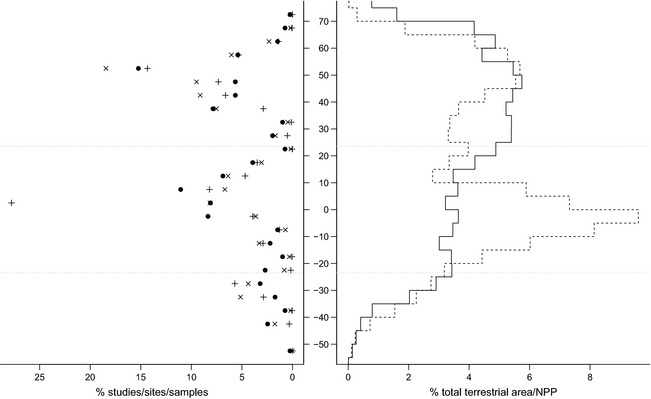
Latitudinal coverage. The percentage of Studies (circles), Sites (crosses) and samples (pluses) in five‐degree bands of latitude. We computed each Study's latitude as the median of its Sites’ latitudes. The solid and dashed lines show the percentage of total terrestrial area and percentage of total terrestrial NPP, respectively, in each five‐degree band (see “ Biogeographical coverage ” in Methods). The dotted horizontal lines indicate the extent of the tropics.

Of the world's 35 biodiversity hotspots, 25 are represented (Table 1). Hotspots together account for just 16% of the world's terrestrial surface, yet 47.67% of our measurements were taken in hotspots. The vast majority of measurements in hotspots were taken in the Sundaland hotspot (Southeast Asia) and the latitudinal band with the most samples is 0° to 5° N (Fig. 2); many of these data come from two studies of higher plants from Indonesia that between them contribute just 284 sites but over 320,000 samples (Sheil et al. 2002).

**Table 1:** Coverage of hotspots.

Hotspot	Studies (%)	Sites (%)	Samples (%)	Terrestrial area (%)
None	50.72	63.63	52.33	84.01
Nearctic				
California Floristic Province	0.96	1.30	0.12	0.20
Madrean Pine–Oak Woodlands	0.24	0.01	<0.01	0.31
Neotropic				
Atlantic Forest	3.11	1.16	0.28	0.83
Caribbean Islands	0.48	0.67	2.59	0.15
Cerrado	1.91	0.66	0.11	1.37
Chilean Winter Rainfall and Valdivian Forests	2.39	1.69	0.32	0.27
Mesoamerica	8.13	7.83	8.94	0.76
Tropical Andes	6.46	3.02	4.11	1.04
Tumbes‐Choco‐Magdalena	0.48	0.37	0.10	0.18
Palearctic				
Caucasus	0.00	0.00	0.00	0.36
Irano‐Anatolian	0.00	0.00	0.00	0.61
Japan	1.67	0.60	0.17	0.25
Mediterranean Basin	5.98	5.52	2.63	1.41
Mountains of Central Asia	0.00	0.00	0.00	0.58
Mountains of Southwest China	0.00	0.00	0.00	0.18
Afrotropic				
Cape Floristic Region	0.24	0.29	0.20	0.05
Coastal Forests of Eastern Africa	0.00	0.00	0.00	0.20
Eastern Afromontane	1.20	1.27	0.83	0.07
Guinean Forests of West Africa	2.15	1.04	0.54	0.42
Horn of Africa	0.00	0.00	0.00	1.12
Madagascar and the Indian Ocean Islands	0.48	0.18	0.01	0.40
Maputaland–Pondoland–Albany	0.72	0.52	0.50	0.18
Succulent Karoo	0.00	0.00	0.00	0.07
Indo‐Malay				
Himalaya	0.00	0.00	0.00	0.50
Indo‐Burma	0.72	0.23	0.10	1.60
Philippines	1.20	0.77	0.44	0.20
Sundaland	6.46	6.12	23.55	1.01
Western Ghats and Sri Lanka	0.48	0.13	0.09	0.13
Australasia				
East Melanesian Islands	0.24	0.36	1.13	0.68
Forests of East Australia	0.72	1.45	0.31	0.17
New Caledonia	0.00	0.00	0.00	0.01
New Zealand	0.72	0.10	0.01	0.18
Southwest Australia	0.00	0.00	0.00	0.24
Wallacea	1.67	0.69	0.58	0.23
Oceania				
Polynesia–Micronesia	0.48	0.38	0.01	0.03

Hotspots are shown grouped by realm.

**Figure 3:**
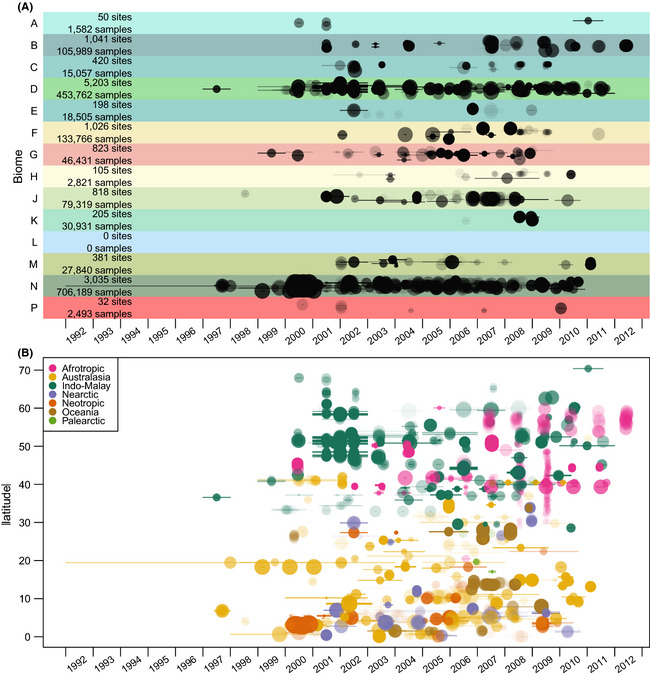
Spatiotemporal sampling coverage. Site sampling dates by biome (A) and absolute latitude (B). Each Site is represented by a circle and line. Circle radii are proportional to log_10_ of the number of samples at that Site. Circle centers are at the midpoints of Site sampling dates; lines indicate the start and end dates of sampling. Y‐values in (A) have been jittered at the study level. Circles and lines have the same degree of partial transparency. Biome colors and letters in (A) are as in Fig. 1. Colors in (B) indicate biogeographic realm.

**Figure 4:**
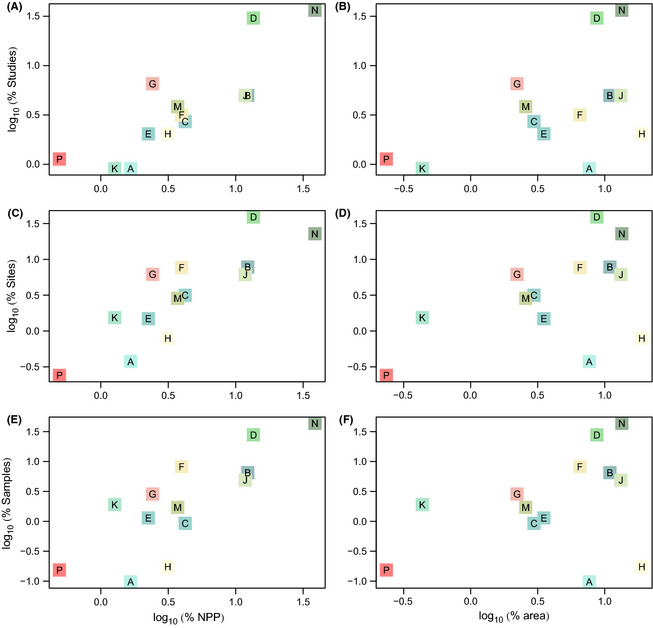
Coverage of biomes. The percentage of Studies (A and B), Sites (C and D) and samples (E and F) against percentages of terrestrial NPP (A, C and E) and terrestrial area (B, D and F). Biome colors and letters are as in Fig. 1.

The best‐represented biomes are “Temperate Broadleaf and Mixed Forests” and “Tropical and Subtropical Moist Broadleaf Forests” (Figs 3, 4). “Flooded Grasslands and Savannas” is the only biome that is unrepresented in our database (Figs 3, 4); although this biome is responsible for only 0.7% of global terrestrial net primary productivity, it is nevertheless ecologically important and will be a priority for future collation efforts. Two biomes – “Tundra” and “Deserts and Xeric Shrublands” – are underrepresented relative to their areas. Of the world's 17 megadiverse countries identified by Mittermeier et al. (1997), only Democratic Republic of Congo is not represented (Figure S4). The vast majority of sampling took place after the year 2000 (Fig. 3), reflecting our desire to collate diversity data that can be related to MODIS data, which are available from early 2000 onwards. The database's coverage of realms, biomes, countries, regions and subregions is shown in Supplementary Tables S5–S11.

**Figure 5:**
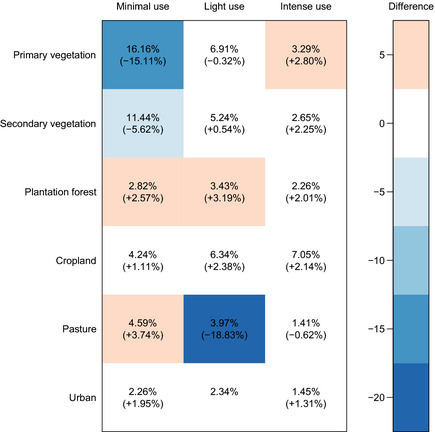
Representativeness of predominant land use and land‐use intensity classes. Numbers are the percentage of Sites assigned to each combination of land use and intensity. Numbers in brackets and colors are the differences between these and the proportional estimated total terrestrial area of each combination of land use and land‐use intensity for 2005, computed from the HYDE (Hurtt et al. 2011) and Global Land Systems datasets (van Asselen and Verburg 2012); no difference is shown for “Urban”/”Light use” because these datasets did not allow us to compute an estimate for this combination. The 12.15% of Sites that could not be assigned a classification for predominant land use and/or land‐use intensity are not shown.

The distribution of Site‐level predominant land use and use intensity is different from the distribution of the estimated total terrestrial area in each land use/land‐use intensity combination for 2005 (*χ*2 = 28,243.21, df = 16, *P* < 2.2 × 10^−16^; we excluded “Urban”/”Light use” from this test because the HYDE and Global Land Systems datasets did not allow us to compute an estimate for this combination). The main discrepancies are that the database has far fewer than expected Sites that are classified as “Primary habitat”/“Minimal use”, “Secondary vegetation”/“Light use” and “Pasture”/“Light use” (Fig. 5). We were unable to assign a classification of predominant land use to 3.34% of Sites and of use intensity to 12.09% of Sites. The most common fragmentation layout was “Representative part of a fragmented landscape” (27.95% of Sites; Table S12) – a classification that indicates either that a Site is large enough to encompass multiple habitat types or that the Site is of a particular habitat type that is inherently fragmented and dominates the landscape e.g., the site is in an agricultural field and the landscape is comprised of many fields. We were unable to assign a fragmentation layout to 15.47% of Sites. We were able to determine the maximum linear extent of sampling for 60.09% of Sites – values range from 0.2 m to 39.15 km; median 120 m (Figure S5). The precise sampling days are known for 45.44% of Sites; 42.19% are known to the nearest month and 12.37% to the nearest year. The median sampling duration was 91 days; sampling lasted for 1 day or less at 9.90% of Sites (Figure S6). The area of habitat containing the site is known for 25.49% of Sites – values are approximately log‐normally distributed (median 40,000 square meters; Figure S7). We reviewed all cases of Sites falling outside the GIS polygons for countries (0.82% of Sites; Figure S8) and ecoregions (0.52% of Sites; Figure S9). These Sites were either on coasts and/or on islands too small to be included in the GIS dataset in question.

**Figure 6:**
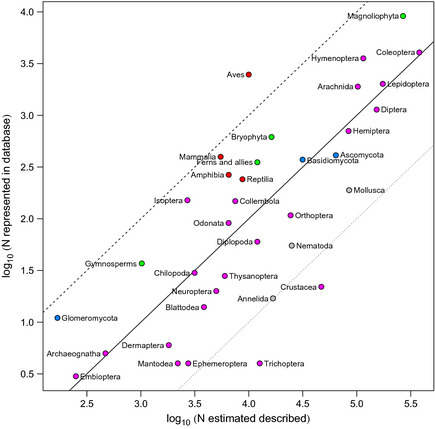
Taxonomic coverage. The number of species in our database against the number of described species as estimated by Chapman (2009). Vertebrates are shown in red, arthropods in pink, other animals in gray, plants in green and fungi in blue. The dashed, solid and dotted lines indicate 10, 1 and 0.1% representation, respectively. Groups with just a single species in the database – Diplura, Mycetozoa, Onychophora, Pauropoda, Phasmida, Siphonaptera, Symphyla and Zoraptera – are not shown.

The database contains measurements of approximately 28,735 species (see “ Counting the number of species ” in Methods) – 17,733 animals, 10,201 plants, 800 fungi and 1 protozoan. We were unable to place 97 taxa in a higher taxonomic group because they were not sufficiently well resolved. The database contains more than 1% as many species as have been described within 20 higher taxonomic groups (Fig. 6). Birds are particularly well represented, reflecting the sampling bias in favor of this charismatic group. Our database contains measurements of 2,479 species of birds – 24.81% of those described (Chapman 2009) – and 2,368 of these are resolved to either species or infraspecies levels. A total of 228,644 samples – more than 14% of the entire database – are of birds. In contrast, just 397 species of mammals are represented, but even this constitutes 7.24% of described species. Chiroptera (bats) are the best‐represented mammalian order with 188 species. Of the 115,000 estimated described species of Hymenoptera, 3,556 (3.09%) are represented in the database, the best representation of an invertebrate group. The hymenopteran family with the most species in the database is Formicidae with 2,060 species. The database contains data for 4,056 species of Coleoptera – 1.07% of described beetles. Carabidae is the best‐represented beetle family with 2,060 species. Some higher taxonomic groups have well below 1% representation and, as might be expected, the database has poor coverage of groups for which the majority of species are marine – nematodes, crustaceans and molluscs.

**Figure 7:**
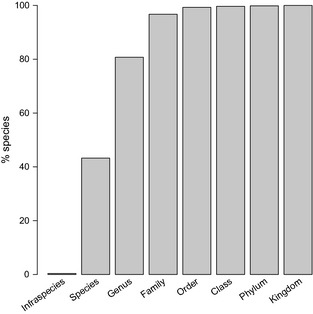
Cumulative percentage of species in the database, by the taxonomic rank at which the name was matched to COL.

**Figure 8:**
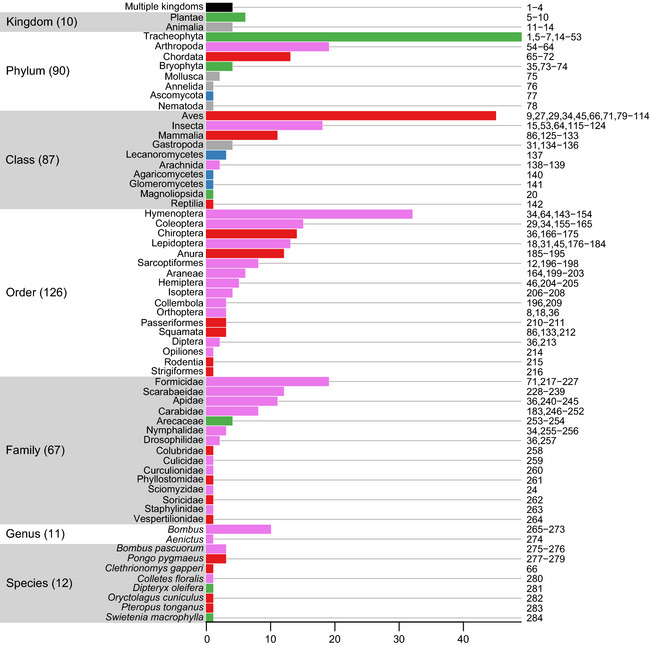
Number of Studies by lowest common taxonomic group. Bars show the number of Studies within each lowest common taxon (so, one Study examined the species *Swietenia macrophylla*, three Studies examined the species *Bombus pascuorum*, ten Studies examined multiple species within the genus *Bombus*, and so on). Colors are as in Figure 6. Numbers on the right are the primary references from which data were taken: 1 López‐Quintero et al. 2012; 2 Buscardo et al. 2008; 3 Domínguez et al. 2012; 4 Nöske et al. 2008; 5 Center for International Forestry Research (CIFOR) 2013a; 6 Center for International Forestry Research (CIFOR) 2013b; 7 Sheil et al. 2002; 8 Dumont et al. 2009; 9 Proenca et al. 2010; 10 Baeten et al. 2010a, b; 11 Richardson et al. 2005; 12 Schon et al. 2011; 13 Muchane et al. 2012; 14 Vázquez and Simberloff 2002; 15 Bouyer et al. 2007; 16 O'Connor 2005; 17 Higuera and Wolf 2010; 18 Kati et al. 2012; 19 Lucas‐Borja et al. 2011; 20 Louhaichi et al. 2009; 21 Power et al. 2012; 22 Brearley 2011; 23 Baeten et al. 2010a; 24 Williams et al. 2009; 25 Mayfield et al. 2006; 26 Kolb and Diekmann 2004; 27 Phalan et al. 2011; 28 Vassilev et al. 2011; 29 Paritsis and Aizen 2008; 30 Boutin et al. 2008; 31 Baur et al. 2006; 32 Fensham et al. 2012; 33 Brunet et al. 2011; 34 Kessler et al. 2009; 35 Hylander and Nemomissa 2009; 36 Barlow et al. 2007; 37 Kumar and Shahabuddin 2005; 38 Kessler et al. 2005; 39 Hietz 2005; 40 Krauss et al. 2004; 41 Hernández et al. 2012; 42 Calviño‐Cancela et al. 2012; 43 Golodets et al. 2010; 44 Castro et al. 2010; 45 Milder et al. 2010; 46 Helden and Leather 2004; 47 McNamara et al. 2012; 48 Katovai et al. 2012; 49 Berry et al. 2010; 50 Letcher and Chazdon 2009; 51 Romero‐Duque et al. 2007; 52 Marin‐Spiotta et al. 2007; 53 Power and Stout 2011; 54 Norfolk et al. 2012; 55 Poveda et al. 2012; 56 Cabra‐García et al. 2012; 57 Turner and Foster 2009; 58 Woodcock et al. 2007; 59 Lachat et al. 2006; 60 Rousseau et al. 2013; 61 Nakamura et al. 2003; 62 Basset et al. 2008; 63 Hanley 2011; 64 Billeter et al. 2008; Diekötter et al. 2008; Le Féon et al. 2010; 65 Sung et al. 2012; 66 St‐Laurent et al. 2007; 67 Centro Agronómico Tropical de Investigación y Enseñanza (CATIE) 2010; 68 Endo et al. 2010; 69 Alcala et al. 2004; 70 Bicknell and Peres 2010; 71 Woinarski et al. 2009; 72 Garden et al. 2010; 73 Hylander and Weibull 2012; 74 Giordano et al. 2004; 75 Ström et al. 2009; 76 Römbke et al. 2009; 77 Giordani 2012; 78 Hu and Cao 2008; 79 Edenius et al. 2011; 80 O'Dea and Whittaker 2007; 81 Ims and Henden 2012; 82 Rosselli 2011; 83 Arbeláez‐Cortés et al. 2011; 84 Santana et al. 2012; 85 Sheldon et al. 2010; 86 Wang et al. 2010; 87 Sodhi et al. 2010; 88 Naoe et al. 2012; 89 Cerezo et al. 2011; 90 Lantschner et al. 2008; 91 Chapman and Reich 2007; 92 Báldi et al. 2005; 93 Farwig et al. 2008; 94 Shahabuddin and Kumar 2007; 95 Borges 2007; 96 Wunderle et al. 2006; 97 Politi et al. 2012; 98 Moreno‐Mateos et al. 2011; 99 Mallari et al. 2011; 100 Latta et al. 2011; 101 Sosa et al. 2010; 102 Miranda et al. 2010; 103 Flaspohler et al. 2010; 104 Bóçon 2010; 105 Azpiroz and Blake 2009; 106 Aben et al. 2008; 107 Cockle et al. 2005; 108 Vergara and Simonetti 2004; 109 Azhar et al. 2013; 110 Reid et al. 2012; 111 Neuschulz et al. 2011; 112 Dawson et al. 2011; 113 Naidoo 2004; 114 Dures and Cumming 2010; 115 Meyer et al. 2009; 116 Summerville 2011; 117 Cleary et al. 2004; 118 Mudri‐Stojnic et al. 2012; 119 Schüepp et al. 2011; 120 Bates et al. 2011; 121 Quintero et al. 2010; 122 Vergara and Badano 2009; 123 Kohler et al. 2008; 124 Meyer et al. 2007, 125 Hoffmann and Zeller 2005; 126 Caceres et al. 2010; 127 Lantschner et al. 2012; 128 Wells et al. 2007; 129 Bernard et al. 2009; 130 Martin et al. 2012; 131 Gheler‐Costa et al. 2012; 132 Sridhar et al. 2008; 133 Scott et al. 2006; 134 Oke 2013; 135 Oke and Chokor 2009; 136 Kappes et al. 2012; 137 Walker et al. 2006; 138 Lo‐Man‐Hung et al. 2008; 139 Zaitsev et al. 2002; 140 Robles et al. 2011; 141 Brito et al. 2012; 142 Luja et al. 2008; 143 Smith‐Pardo and Gonzalez 2007; 144 Schüepp et al. 2012; 145 Tylianakis et al. 2005; 146 Verboven et al. 2012; 147 Osgathorpe et al. 2012; 148 Tonietto et al. 2011; 149 Samnegård et al. 2011; 150 Cameron et al. 2011; 151 Malone et al. 2010; 152 Marshall et al. 2006; 153 Shuler et al. 2005; 154 Quaranta et al. 2004; 155 Légaré et al. 2011; 156 Noreika 2009; 157 Otavo et al. 2013; 158 Numa et al. 2012; 159 Jonsell 2012; 160 Mico et al. 2013; 161 Rodrigues et al. 2013; 162 Sugiura et al. 2009; 163 Verdú et al. 2007; 164 Banks et al. 2007; 165 Elek and Lovei 2007; 166 Fukuda et al. 2009; 167 Castro‐Luna et al. 2007; 168 Shafie et al. 2011; 169 Struebig et al. 2008; 170 Threlfall et al. 2012; 171 Presley et al. 2008; 172 Willig et al. 2007; 173 MacSwiney et al. 2007; 174 Clarke et al. 2005; 175 Sedlock et al. 2008; 176 Verdasca et al. 2012; 177 D'Aniello et al. 2011; 178 Berg et al. 2011; 179 Summerville et al. 2006; 180 Hawes et al. 2009; 181 Cleary and Mooers 2006; 182 Krauss et al. 2003; 183 Ishitani et al. 2003; 184 Safian et al. 2011; 185 Furlani et al. 2009; 186 Isaacs‐Cubides and Urbina‐Cardona 2011; 187 Gutierrez‐Lamus 2004; 188 Adum et al. 2013; 189 Watling et al. 2009; 190 Pillsbury and Miller 2008; 191 Pineda and Halffter 2004; 192 Ofori‐Boateng et al. 2013; 193 de Souza et al. 2008; 194 Faruk et al. 2013; 195 Hilje and Aide 2012; 196 Alberta Biodiversity Monitoring Institute (ABMI) 2013; 197 Zaitsev et al. 2006; 198 Arroyo et al. 2005; 199 Paradis and Work 2011; 200 Buddle and Shorthouse 2008; 201 Kapoor 2008; 202 Alcayaga et al. 2013; 203 Magura et al. 2010; 204 Littlewood et al. 2012; 205 Kőrösi et al. 2012; 206 Oliveira et al. 2013; 207 Carrijo et al. 2009; 208 Reis and Cancello 2007; 209 Chauvat et al. 2007; 210 Otto and Roloff 2012; 211 Zimmerman et al. 2011; 212 Pelegrin and Bucher 2012; 213 Savage et al. 2011; 214 Bragagnolo et al. 2007; 215 Jung and Powell 2011; 216 Bartolommei et al. 2013; 217 Dominguez‐Haydar and Armbrecht 2010; 218 Armbrecht et al. 2006; 219 Hashim et al. 2010; 220 Schmidt et al. 2012; 221 Maeto and Sato 2004; 222 Bihn et al. 2008; 223 Delabie et al. 2009; 224 Fayle et al. 2010; 225 Gove et al. 2005; 226 Buczkowski and Richmond 2012; 227 Buczkowski 2010; 228 Noriega et al. 2012; 229 Navarro et al. 2011; 230 Noriega et al. 2007; 231 Horgan 2009; 232 Gardner et al. 2008; 233 da Silva 2011; 234 Silva et al. 2010; 235 Jacobs et al. 2010; 236 Slade et al. 2011; 237 Filgueiras et al. 2011; 238 Navarrete and Halffter 2008; 239 Davis and Philips 2005; 240 Parra‐H and Nates‐Parra 2007; 241 Fierro et al. 2012; 242 Nielsen et al. 2011; 243 Julier and Roulston 2009; 244 Winfree et al. 2007; 245 Hanley 2005; 246 Liu et al. 2012; 247 Gu et al. 2004; 248 Noreika and Kotze 2012; 249 Rey‐Velasco and Miranda‐Esquivel 2012; 250 Vanbergen et al. 2005; 251 Koivula et al. 2004; 252 Weller and Ganzhorn 2004; 253 Carvalho et al. 2010; 254 Aguilar‐Barquero and Jiménez‐Hernández 2009; 255 Fermon et al. 2005; 256 Ribeiro and Freitas 2012; 257 Gottschalk et al. 2007; 258 Cagle 2008; 259 Johnson et al. 2008; 260 Su et al. 2011; 261 Saldana‐Vazquez et al. 2010; 262 Nicolas et al. 2009; 263 Sakchoowong et al. 2008; 264 Yoshikura et al. 2011; 265 Hanley et al. 2011; 266 Connop et al. 2011; 267 Redpath et al. 2010; 268 Goulson et al. 2010; 269 Goulson et al. 2008; 270 Hatfield and LeBuhn 2007; 271 McFrederick and LeBuhn 2006; 272 Diekötter et al. 2006; 273 Darvill et al. 2004; 274 Matsumoto et al. 2009; 275 Knight et al. 2009; 276 Herrmann et al. 2007; 277 Ancrenaz et al. 2004; 278 Felton et al. 2003; 279 Knop et al. 2004; 280 Davis et al. 2010; 281 Hanson et al. 2008; 282 Ferreira and Alves 2005; 283 Luskin 2010; 284 Grogan et al. 2008.

Of the 28,735 species, 43.26% are matched to a COL record with a rank of species or infraspecies, 37.47% to a COL record with a rank of genus and 19.27% to a COL record with a higher taxonomic rank (Fig. 7). The species with the largest number of measurements – 1,305 – is *Bombus pascuorum* (the common carder bee), and bees constitute 35 of the top 100 most frequently sampled species: this results from a PREDICTS subproject that is examining pollinators. Birds make up most of the remaining top 100, with 36 species. Of the 407 Studies, 126 sampled within a single order (Fig. 8); just 12 Studies examined a single species. The six most commonly examined higher taxonomic groups are Tracheophyta (12.04% of Studies), Aves (11.06%), Hymenoptera (7.86%), Arthropoda (4.67%), Formicidae (4.67%) and Insecta (4.42%). The database contains 17,802 unique values of “Best guess binomial”. The overlap with species attribute databases is often much higher than would be expected by chance (Table 2), greatly facilitating analyses that integrate PREDICTS data with species attributes (Newbold et al. 2013, 2014a).

**Table 2:** Names represented in species attribute databases.

Attribute database	Trait	Group	Best guess binomials	Attribute database names	Species matches	Genus matches	Total matches
GBIF	Range size	All taxa	17,801		14,514		14,514
IUCN	Red list status	All taxa	17,801		3,521		3,521
CITES	CITES appendix	All taxa	17,801	20,094	467		467
PanTHERIA	Body mass	Mammalia	376	3,542	310	62	372
TRY	Seed mass	Plantae	6,924	26,107	2,017	2,820	4,837
TRY	Vegetative height	Plantae	6,924	2,822	772	768	1,540
TRY	Generative height	Plantae	6,924	9,911	1,633	2,546	4,179

GBIF (Global Biodiversity Information Facility, http://www.gbif.org/, queried 2014‐03‐31), IUCN (International Union for Conservation of Nature, http://www.iucn.org/, queried 2014‐03‐31), CITES (Convention on International Trade in Endangered Species of Wild Fauna and Flora, http://www.cites.org/, downloaded 2014‐01‐27), PanTHERIA (Jones et al. 2009), TRY (Kattge et al. 2011). Best guess binomials: the number of unique “Best guess binomials” in the PREDICTS database within that taxonomic group. Attribute database names: the number of unique binomials and trinomials for that attribute in attribute database. Species matches: the number of “Best guess binomials” that exactly match a record in the attribute database. Genus matches: the number of generic names in the PREDICTS database with a matching record in the attribute database (only for binomials for which there was not a species match). Total matches: sum of species matches and genus matches. We did not match generic names for GBIF range size, IUCN category or CITES appendix because we did not expect these traits to be highly conserved within genera.

**Figure 9:**
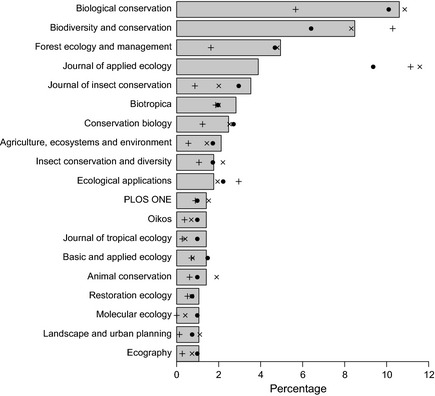
Data contributions by journal. The percentage of Data Sources (bars), Studies (circles), Sites (crosses) and samples (pluses) taken from each journal. Only journals from which more than one Data Source was taken are shown.

Of the 284 Data Sources, 271 were taken from articles published in scientific peer‐reviewed journals; the rest came from unpublished data (5), internet databases (3), PhD theses (2), agency reports (1) and other sources (2). The vast majority – 273 (96.13%) – of Data Sources are taken from English articles; the remainder are in Mandarin (0.35%), Portuguese (1.06%) or Spanish (2.46%). 29.15% of Data Sources come from just four journals (Fig. 9): Biological Conservation (11.07%), Biodiversity and Conservation (8.86%), Forest Ecology & Management (5.17%) and Journal of Applied Ecology (4.06%). The Journal of Applied Ecology contributed many more Studies, Sites and samples than expected from the number of Data Sources (Fig. 9) because of a single Data Source that contributed 21 pan‐European Studies and over 140,000 samples (data taken from Billeter et al. 2008; Diekötter et al. 2008 and Le Féon et al. 2010).

## Discussion

The coverage of the PREDICTS dataset illustrates the large number of published articles that are based on local‐scale empirical data of the responses of diversity either to a difference in land‐use type or along a gradient of land‐use intensity or other human pressure. Such data can be used to model spatial responses of local communities to anthropogenic pressures and thus changes over time. This is essential for understanding the impact of biodiversity loss on ecosystem function and ecosystem services, which operate at the local level (Fontaine et al. 2006; Isbell et al. 2011; Cardinale et al. 2012; Hooper et al. 2012). Regardless of scale, no single Study is or could ever be representative, but the sheer number and diversity of Studies means that a collation of these data can provide relatively representative coverage of biodiversity. The majority of Data Sources (271 of 284) come from peer‐reviewed publications and all data have used peer‐reviewed sampling procedures. There are doubtless very many more published data than we have so far acquired and been given permission to use. For the majority of Data Sources (225), it was necessary to contact the author(s) in order to get more information such as the Site coordinates or the names of the taxa studied: even now that supplementary data are commonplace and often extensive, we usually had to request more detail than had been published.

The database currently lacks Sites in ten biodiversity hotspots and one megadiverse country (Democratic Republic of the Congo). It also has no data from many large tropical or partially tropical countries such as Angola, Tanzania and Zambia. Many countries are underrepresented given their area and/or the distinctiveness of their biota e.g., Australia, China, Madagascar, New Zealand, Russia and South Africa. We have few data from islands and just 57 Sites from the biogeographic realm of Oceania (Fig. 3 and Table S8): we have not yet directly targeted Oceania or island biota more generally. The database contains no studies of microbial diversity and few of parasites – major shortcomings that also apply to other large biodiversity databases such as the Living Planet Index (WWF International 2012), the IUCN Red List (International Union for Conservation of Nature 2013) and BIOFRAG (Pfeifer et al. 2014). Fewer than 50% of the taxa in our database are matched to a Catalogue of Life record with a rank of species or infraspecies (Fig. 6). The quality and coverage of taxonomic databases continues to improve and we hope to improve our database's coverage by making use of new Catalogue of Life checklists as they become available. Improved software would permit the use of fuzzy searches to reduce the current manual work required to curate taxonomic names.

Intersecting our data with datasets of species attributes (Table 2) indicates much greater overlap among large‐scale data resources than might be expected simply based on overall numbers of species. This suggests that the same species are being studied for different purposes, because of either ubiquity, abundance, interest or location. In one sense this is useful, allowing a thorough treatment of certain groups of species, for example by incorporating trait data in analyses. On the other hand, it highlights the fact that many species are poorly studied in terms of distribution, traits and responses to environmental change. Indeed, many taxonomic groups that matter greatly for ecosystem functions (e.g., earthworms, fungi) are routinely underrepresented in data compilations (Cardoso et al. 2011; Norris 2012), including – despite our efforts toward representativeness – ours.

The PREDICTS database is a work in progress, but already represents the most comprehensive database of its kind of which we are aware. Associated with this article is a site‐level extract of the data: columns are described in Table S13. The complete database will be made publicly available in 2015, before which we will attempt to improve all aspects of its coverage by targeting underrepresented hotspots, realms, biomes, countries and taxonomic groups. In addition to taking data from published articles, we will integrate measurements from existing large published datasets, where possible. We welcome and greatly value all contributions of suitable data; please contact us at enquiries@predicts.org.uk.

## Conflict of Interest

None declared.

## Supporting information


**Figure S1.** Maximum linear extents of sampling.
**Figure S2.** Graphical representations of fragmentation layouts.
**Figure S3.** Database schema.
**Figure S4.** Countries represented by area.
**Figure S5.** Histogram of Site maximum linear‐extents of sampling.
**Figure S6.** Histogram of Site sampling durations.
**Figure S7.** Histogram of the area of habitat surrounding each Site.
**Figure S8.** Histogram of the distance from each Site to the nearest country GIS polygon.
**Figure S9.** Histogram of the distance from each Site to the nearest ecoregion GIS polygon.
**Table S1.** Classification of land‐use intensity for primary and secondary vegetation based on combinations of impact level and spatial extent of impact.
**Table S2.** Combinations of predominant land use and use intensity.
**Table S3.** Habitat fragmentation classifications.
**Table S4.** Examples of parsing different styles of taxonomic name with the Global Names Architecture's biodiversity package (https://github.com/GlobalNamesArchitecture/biodiversity).
**Table S5.** Coverage of countries.
**Table S6.** Coverage of regions.
**Table S7.** Coverage of subregions.
**Table S8.** Coverage of realms.
**Table S9.** Coverage of biomes.
**Table S10.** Distribution of samples by biome and kingdom.
**Table S11.** Distribution of samples by subregion and kingdom.
**Table S12.** Coverage of fragmentation layouts.
**Table S13.** Data extract columns.

**Data S1.** Data extract.
