# CORRIGENDUM

**DOI:** 10.1002/ece3.10279

**Published:** 2023-07-20

In the recent article by Hudson et al. (2014), the authors would like to update the URL for the PREDICTS project in the Abstract, Introduction and Discussion sections to read as follow:

https://www.nhm.ac.uk/our‐science/our‐work/biodiversity/predicts.html

The article has been corrected online.
