# Supplementary material for: The PREDICTS database: a global database of how local terrestrial biodiversity responds to human impacts
Source: Ecol Evol. 2014 Dec 2;4(24):4701–35. doi: 10.1002/ece3.1303 (PMC4278822; doi:10.1002/ece3.1303)
Supplement: Supplementary file 1 — Figure S1. Maximum linear extents of sampling. Figure S2. Graphical representations of fragmentation layouts. Figure S3. Database schema. Figure S4. Countries represented by area. Figure S5. Histogram of Site maximum linear‐extents of sampling. Figure S6. Histogram of Site sampling durations. Figure S7. Histogram of the area of habitat surrounding each Site. Figure S8. Histogram of the distance from each Site to the nearest country GIS polygon. Figure S9. Histogram of the distance from each Site to the nearest ecoregion GIS polygon. Table S1. Classification of land‐use intensity for primary and secondary vegetation based on combinations of impact level and spatial extent of impact. Table S2. Combinations of predominant land use and use intensity. Table S3. Habitat fragmentation classifications. Table S4. Examples of parsing different styles of taxonomic name with the Global Names Architecture's biodiversity package (https://github.com/GlobalNamesArchitecture/biodiversity). Table S5. Coverage of countries. Table S6. Coverage of regions. Table S7. Coverage of subregions. Table S8. Coverage of realms. Table S9. Coverage of biomes. Table S10. Distribution of samples by biome and kingdom. Table S11. Distribution of samples by subregion and kingdom. Table S12. Coverage of fragmentation layouts. Table S13. Data extract columns. [file ECE3-4-4701-s002.docx]

The PREDICTS database: a global database of how local terrestrial biodiversity responds to human impacts - supplementary information

| A single trap (e.g., pitfall, light traps, mist net) or quadrat, or single point count | 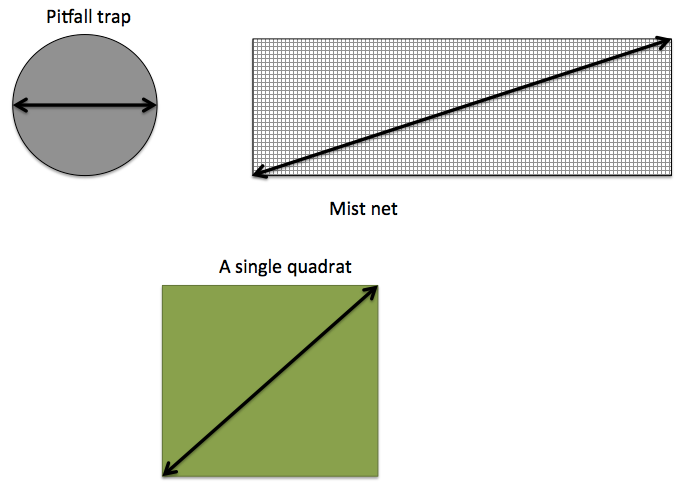 |
| --- | --- |
| Multiple quadrats (randomly placed) | 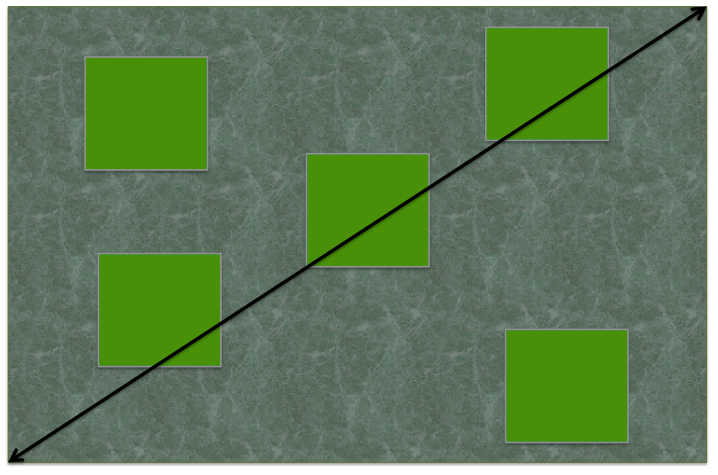  The maximum linear extent is then the diagonal of the whole plot because there is no way of knowing where the quadrats were placed within that space. |
| Multiple quadrats (non-randomly placed) | 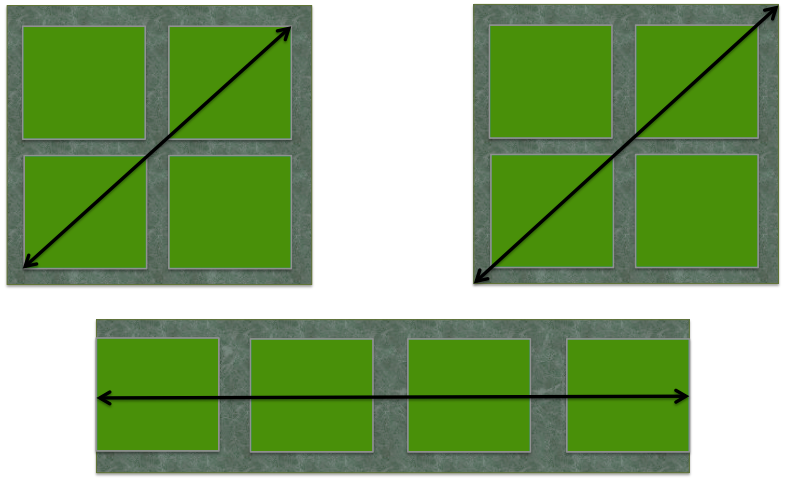  The choice of which to use depends upon the information given in the paper. Use the top-left where we know the distance of the quadrats from each other and can calculate the exact distance that they cover. Use the top-right where we know the total area of the plot and we know that quadrats were placed in a specific manner but do not know how far apart the quadrats were from each other. |
| Multiple traps (ie pitfall, snap traps, light traps) or multiple point counts | 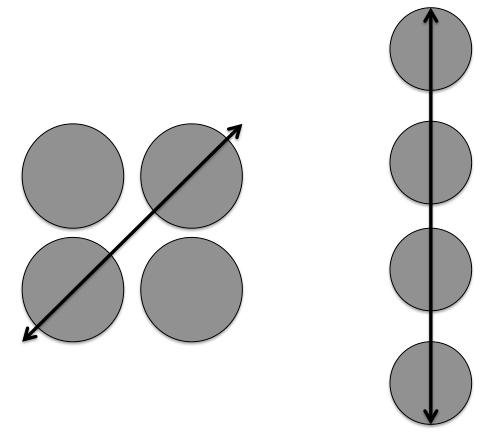 |
| Multiple mist nets | 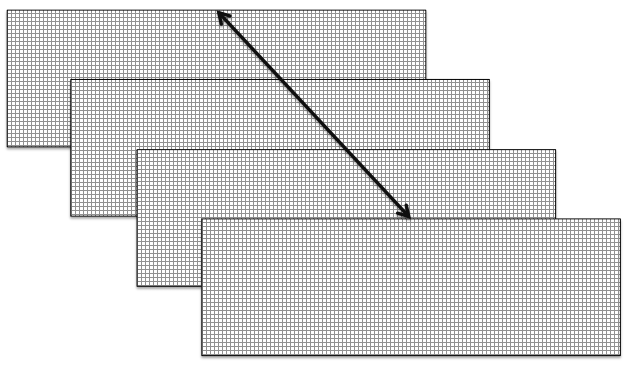 |
| A single transect | 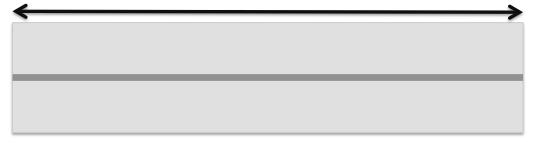 |
| Multiple transects | 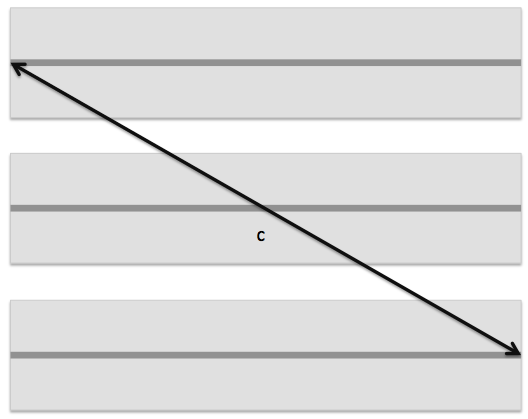  Where $c=\sqrt{{(length of transect)}^{2}+{(distance between transects)}^{2}}$ |

Supplementary Figure S.1. Maximum linear extents of sampling.

The thick black line indicates the distance that was recorded.

**Notes on assigning predominant land use and use intensity**

Please bear in mind that most studies that compare biodiversity at multiple sites are not relevant to PREDICTS, because the sites do not vary meaningfully in the nature and intensity of human impacts that they face, or because the variation cannot be captured within the PREDICTS framework. Categories of studies that are not relevant include surveys unlinked to anthropogenic threats, comparisons along natural gradients and comparisons of subtly different management regimes. Please try to make sure that a study’s data is likely to be of value to PREDICTS before asking authors for the data or processing data from the paper.

**What does Predominant Land Use mean?**

Each site’s biodiversity data come from samples taken within the sampling frame. The sampling frame might be (among other possibilities) a quadrat, a transect or a polygon within which an array of sampling points are placed. The physical size of the sampling frame (which is captured by the “Maximum Linear Extent Sampled” field within the Excel file) is likely to differ among studies and to depend on the taxonomic group being sampled. The site’s Predominant Land Use is the land-use class that best describes the land use in the sampling frame or, if the Maximum Linear Extent Sampled is less than 10m, the 100m^2^ centred around the sampling frame. The exception to this is urban sites: sites that are within built-up areas can be classed as urban even if they are in large urban parks (see below).

Some ways people use the land are hybrids between classes listed here. For example, agroforestry combines Plantation Forest and Cropland, while wood-pasture combines Plantation Forest and Pasture. If the description of a site indicates which aspect of a mixture of land uses is dominant, classify the site accordingly. Otherwise, assess the Use Intensity under each of the two classes and classify the site’s Predominant Land Use in the land-use class that has the higher Use-Intensity**.**

Few papers classify land use in exactly the way PREDICTS does, and site descriptions are not always sufficiently clear that you will be able to classify the site with confidence. If you cannot confidently classify a site’s Predominant Land Use, class it as CANNOT DECIDE.

**Definitions of Predominant Land Use classes**

PRIMARY vegetation (forest or non-forest) is native vegetation that is not known or inferred to have ever been completely destroyed, before the year in which the biodiversity was sampled, by human actions or by extreme natural events that do not normally play a role in ecosystem dynamics. Sites where primary vegetation has been destroyed by natural events that are part of the normal ecosystem dynamic (e.g. fire in Mediterranean ecosystems) remain as primary vegetation provided that colonization from adjacent habitat and regeneration is possible. Sites in urban and suburban settings where the vegetation has never been completely destroyed should also be classed as primary vegetation. Synonyms include “ancient woodland”, “old-growth forest” and “natural grassland” (unless any indication is given of a previous land use class). Primary vegetation can be used by people (e.g., fruit harvesting, selective logging). Primary vegetation includes sites where people have tried to restore degraded habitat, so long as the native vegetation has never been destroyed. Sites where the original vegetation is known to have been completely destroyed should not be classed as primary vegetation.

The decision whether to classify Primary sites as Primary Forest or Primary Non-Forest is not important for analysis (they are pooled), but should reflect the descriptions in the paper.

SECONDARY vegetation is where the original primary vegetation was completely destroyed. This could be by human actions (including fire), and includes where sites are recovering to a natural state following a period of human-dominated land use (cropland, plantation forest, pasture or urban). Also counted as secondary are places where natural events (fires, storms etc.) have destroyed the vegetation, but not where the vegetation is naturally maintained by fire (such as climatically Mediterranean systems), which would be primary. Secondary vegetation includes areas where humans have made an active attempt (through planting etc.) to return an area where the natural vegetation was previously destroyed to a more natural state. Synonyms include “old-field”, “abandoned” and “fallow”. Although not managed as intensively as the human-dominated classes, such sites can be used by people in much the same way as primary vegetation sites.

The decision whether to classify Secondary sites as Young Secondary, Intermediate Secondary, Mature Secondary or Secondary (Indeterminate) should depend on structural complexity of the vegetation as described in the paper, with the time since the site became secondary vegetation being a reasonable proxy (though ongoing use not sufficient to destroy the vegetation might nevertheless prevent vegetation from reaching the structural complexity that this time might suggest). Young Secondary Vegetation has a simple architecture representing an early successional stage; forest stands less than 10 years old in the tropics or 30 years old in temperate regions would likely fall into this category, as do sites where the primary vegetation has only just been destroyed (e.g., by site-wide clear-felling). Intermediate Secondary Vegetation has a mixed architecture showing a mid-successional stage, roughly corresponding to stands aged 10-30 years in the tropics or 30-75 years in temperate regions. Mature Secondary Vegetation has architectural structure approaching that of primary vegetation, corresponding to a completed succession; such forest stands would typically be at least 30 years old in the tropics or 75 years old in temperate regions. Please note that secondary grassland will be Young Secondary Vegetation except in places that are too dry, too cold, have too little soil, have too much naturally-occurring fire, or have too much natural grazing for forest to develop.

If you are not sure which of two adjacent age categories best describes a site, make a choice between them: we recognise that the boundaries between age categories are to an extent fuzzy. If, however, you have no information at all on the age/stage of a secondary site, classify it as Secondary Vegetation (Indeterminate Age).

It is extremely important to put sites into age classes based on these descriptions, and to avoid any temptation to spread sites across the full range of age classes when differences are slight.

PLANTATION FOREST applies to previously cleared areas that people have planted with crop trees or crop shrubs for commercial or subsistence harvesting of wood and/or fruit. The species planted may or may not be native. Planting an area with native woody plants for habitat restoration rather than for goods does not constitute plantation forest; rather this would be secondary vegetation with the stage dependent on the architectural complexity (see definitions under secondary vegetation). Likewise, natural regrowth is not a plantation forest, even if the regrowth will be harvested. If plantation forest is abandoned, it becomes Secondary vegetation – and may have a greater architectural complexity than the time since abandonment might suggest.

CROPLAND is land that people have planted with herbaceous crops, even if these crops will be fed to livestock once harvested. Sites described as “fields”, “arable”, “ploughed” or “tilled” all qualify as cropland. If cropland is abandoned, including temporary abandonment (i.e. fallow), it becomes Secondary vegetation.

PASTURE is land where livestock is known to be grazed regularly or permanently. The plant species may be predominantly native (as in rangelands) or strongly associated with humans (as in European-style pastures). Land that is planted with a crop for harvesting and *subsequently* feeding to livestock is Cropland, not Pasture. However, if vegetation is planted and livestock are grazed *directly* on this vegetation, then this does count as pasture.

URBAN land is areas with human habitation and/or buildings, where the primary vegetation has been removed, and where such vegetation as is present is predominantly managed for civic or personal amenity. Sites within city parks (even if the parks are extensive such that there are no buildings close to the sampling frame), village greens (unless mainly used for grazing) and gardens are all urban sites, as are patches (of any size) of abandoned or waste ground within built-up areas. Remnants of primary vegetation around which suburbs have developed would however be classed as Primary vegetation, and areas with commercial agricultural production, with commercial woody plantations, or grazing would be classified as Cropland, Plantation forest and Pasture respectively.

**What does Use Intensity mean?**

Both within and among data sets, some sites within a given Predominant Land Use are likely to be more heavily used – and so perhaps more severely impacted – by people. Often, the source papers contain information that indicates which sites are more heavily used and which sites less so – but this information is often not quantitative even within a paper and is certainly hard to compare directly between different papers. PREDICTS therefore only tries to capture information on Use Intensity on a three-point scale.

Information on intensity at the level of the sampling frame is often not available. It is acceptable to classify Use Intensity (but not Predominant Land Use) based on information about the surrounding landscape.

The definitions of use intensity for each Predominant Land Use are given in the matrix below. For primary and secondary vegetation, where the classification of intensity can be based on both the level of human impact and the spatial extent of that impact, you might find Table 1 helpful. The Use Intensity categories are unlikely to have exactly the same biological implications in different Predominant Land Use classes, but are intended to provide some scope for differentiating sites facing few disturbances from sites facing many.

| **Impact** | **Extent of site affected** | | |
| --- | --- | --- | --- |
|  | **Small fraction** | **Large fraction** | **Most/all of site** |
| No definite ongoing influences to habitat architecture OR no severe ongoing threats to any guild explicitly mentioned | Minimal | Minimal | Light |
| Ongoing influences but not fundamental alterations to habitat architecture OR severe ongoing threats to some guilds (but not guilds that define the nature of the ecosystem) | Light | Light | Intense |
| Ongoing fundamental alterations to habitat architecture (but not complete destruction of vegetation) OR ongoing serious threats to keystone guilds OR ongoing serious threats to many guilds | Light | Intense | Intense |

Supplementary Table S1. Classification of land-use intensity for primary and secondary vegetation based on combinations of impact level and spatial extent of impact.

If you are not sure which of two adjacent Use Intensity categories best describes a site, make a choice between them: we recognise that the boundaries between categories are to an extent fuzzy. If, however, you are entirely unable to infer a site’s Use Intensity, please assign CANNOT DECIDE.

It is very important to classify Use Intensity by trying to match descriptions given in the paper with those in Supplementary Table S2: don’t try to spread sites in a paper across as many Use Intensity classes as possible if the differences between them are too small to justify doing so.

| **Level 1 Land Use** | **Predominant Land Use** | **Minimal use** | **Light use** | **Intense use** |
| --- | --- | --- | --- | --- |
| No evidence of prior destruction of the vegetation | Primary forest | Any disturbances identified are very minor (e.g., a trail or path) or very limited in the scope of their effect (e.g., hunting of a particular species of limited ecological importance). | One or more disturbances of moderate intensity (e.g., selective logging) or breadth of impact (e.g., bushmeat extraction), which are not severe enough to markedly change the nature of the ecosystem. Primary sites in suburban settings are at least Light use. | One or more disturbances that is severe enough to markedly change the nature of the ecosystem; this includes clear-felling of part of the site too recently for much recovery to have occurred. Primary sites in fully urban settings should be classed as Intense use. |
|  | Primary Non-Forest | As above | As above | As above |
| Recovering after destruction of the vegetation | Mature Secondary Vegetation | As for Primary Vegetation-Minimal use | As for Primary Vegetation-Light use | As for Primary Vegetation-Intense use |
|  | Intermediate Secondary Vegetation | As for Primary Vegetation-Minimal use | As for Primary Vegetation-Light use | As for Primary Vegetation-Intense use |
|  | Young Secondary Vegetation | As for Primary Vegetation-Minimal use | As for Primary Vegetation-Light use | As for Primary Vegetation-Intense use |
|  | Secondary Vegetation (indeterminate age) | As for Primary Vegetation-Minimal use | As for Primary Vegetation-Light use | As for Primary Vegetation-Intense use |

| **Level 1 Land Use** | **Predominant Land Use** | **Minimal use** | **Light use** | **Intense use** |
| --- | --- | --- | --- | --- |
| Human use (agricultural) | Plantation forest | Extensively managed or mixed timber, fruit/coffee, oil-palm or rubber plantations in which native understorey and/or other native tree species are tolerated, which are not treated with pesticide or fertiliser, and which have not been recently (< 20 years) clear-felled. | Monoculture fruit/coffee/rubber plantations with limited pesticide input, or mixed species plantations with significant inputs. Monoculture timber plantations of mixed age with no recent (< 20 years) clear-felling. Monoculture oil-palm plantations with no recent (< 20 years) clear-felling. | Monoculture fruit/coffee/rubber plantations with significant pesticide input.  Monoculture timber plantations with similarly aged trees or timber/oil-palm plantations with extensive recent (< 20 years) clear-felling. |
| Human use (agricultural) | Cropland | Low-intensity farms, typically with small fields, mixed crops, crop rotation, little or no inorganic fertiliser use, little or no pesticide use, little or no ploughing, little or no irrigation, little or no mechanisation. | Medium intensity farming, typically showing some but not many of the following: large fields, annual ploughing, inorganic fertiliser application, pesticide application, irrigation, no crop rotation, mechanisation, monoculture crop. Organic farms in developed countries often fall within this category, as may high-intensity farming in developing countries. | High-intensity monoculture farming, typically showing many of the following features: large fields, annual ploughing, inorganic fertiliser application, pesticide application, irrigation, mechanisation, no crop rotation. |
|  | Pasture | Pasture with minimal input of fertiliser and pesticide, and with low stock density (*not* high enough to cause significant disturbance or to stop regeneration of vegetation). | Pasture either with significant input of fertiliser or pesticide, or with high stock density (high enough to cause significant disturbance or to stop regeneration of vegetation). | Pasture with significant input of fertiliser or pesticide, *and* with high stock density (high enough to cause significant disturbance or to stop regeneration of vegetation). |
| Human use (urban) | Urban | Extensive managed green spaces; villages. | Suburban (e.g. gardens), or small managed or unmanaged green spaces in cities. | Fully urban with no significant green spaces. |

Supplementary Table S2. Combinations of predominant habitat and use intensity.

| **Category** | **Landscape Level** | **Study Level** | **Site Level** |
| --- | --- | --- | --- |
| **Well within unfragmented habitat** | Habitat is continuous across the landscape – habitat does not need to be primary vegetation (e.g., continuous plantation forests or a continuous secondary forest). |  | Sample site is well within the unfragmented landscape – this is scale dependent, with information being obtained from the paper/author. |
| **Within unfragmented habitat but at or near its edge** | Habitat is continuous across the landscape – habitat does not need to be primary vegetation (e.g., continuous plantation forests or a continuous secondary forest). |  | Sample site is at the edge of the unfragmented landscape – this is scale dependent, with information being obtained from the paper/author. |
| **Within a remnant patch (perhaps at its edge) that is surrounded by other habitats** | Landscape is fragmented with different habitat types – some of which might be primary (natural) vegetation. | Patches are considered older than the matrix surrounding them, and are assumed to have a higher biodiversity value. | Sample site is located in a patch of older, well-established habitat type, (eg. primary vegetation, mature secondary forest).  The site can be at the edge of the patch, and therefore surrounded by other habitat types. |
| **Representative part of a fragmented landscape** | Landscape is fragmented with different habitat types – some of which might be primary (natural) vegetation. Some landscapes are considered inherently fragmented; for example, agricultural and urban landscapes. |  | Either;  Relatively unknown what the site level habitat is like, either due to lack of information from the data or due to the site being large enough to encompass multiple habitat types.  Or;  The sample site is of a particular habitat type that is inherently fragmented, and dominates the landscape e.g., the site is in an agricultural field and the landscape is comprised of many fields. |
| **Part of the matrix surrounding remnant patches** | Landscape is fragmented with different habitat types – some of which might be primary (natural) vegetation. | Patches are considered older than the matrix surrounding them, and are assumed to have a higher biodiversity value.  This will often be used when the study is dealing with fragmentation, and therefore have sites within a more established habitat type and outside of it for comparison. | Site is located outside of the remnant, older habitat type in a more recently established habitat type. Ideally the exact distance will be known and can be entered in the excel spreadsheet. |
| **Cannot decide** | Information not provided. | Information not provided. | Information not provided. |

Supplementary Table S3. Habitat fragmentation classifications.

| **Well within unfragmented habitat** | 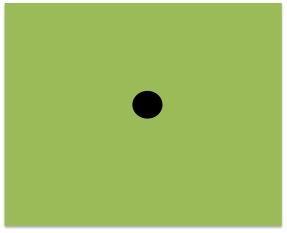  Black dot represents sample site.  Light green represents an unfragmented landscape of any habitat type. |
| --- | --- |
| **Within unfragmented habitat but at or near its edge** | 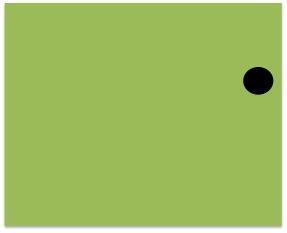  Black dot represents sample site.  Light green represents an unfragmented landscape of any habitat type. |
| **Within a remnant patch (perhaps at its edge) that is surrounded by other habitats** | 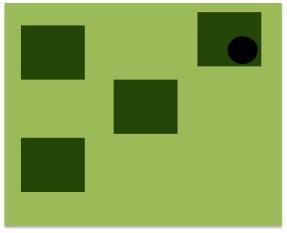  Black dot represents sample site.  Dark green fragments represents a habitat type that is older than surrounding (light green) matrix.  Sample site can be at any position within dark green fragments.  Both greens can be of any habitat type, as long as the fragments can be considered to be of higher biodiversity value. |
| **Representative part of a fragmented landscape** | 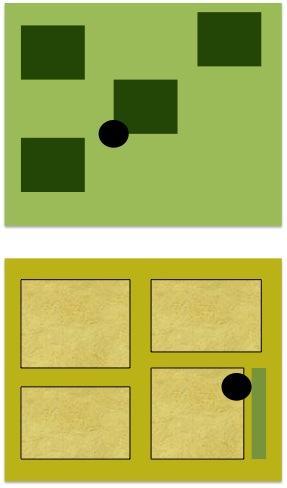  Black dot represents sample site.  Both diagrams depict a fragmented landscape – the top represents a vegetative landscape, and the bottom a more agricultural setting.  Colours don’t necessarily depict habitats of different ages, just that the landscape is fragmented.  Sample site can be anywhere within the fragments, and potentially might overlap multiple habitats.  Some landscapes are considered fragmented by definition – such as an agricultural area - the sample site could be anywhere within the landscape. |
| **Part of the matrix surrounding remnant patches** | 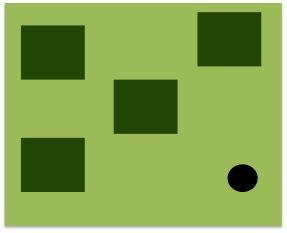  Black dot represents sample site.  Dark green fragments a represents habitat type that is older than surrounding (light green) matrix.  Sample site can be at any position within light green matrix.  Both greens can be of any habitat type, as long as the fragments can be considered to be of higher biodiversity value. |

Supplementary Figure S2. Graphical representations of fragmentation layouts
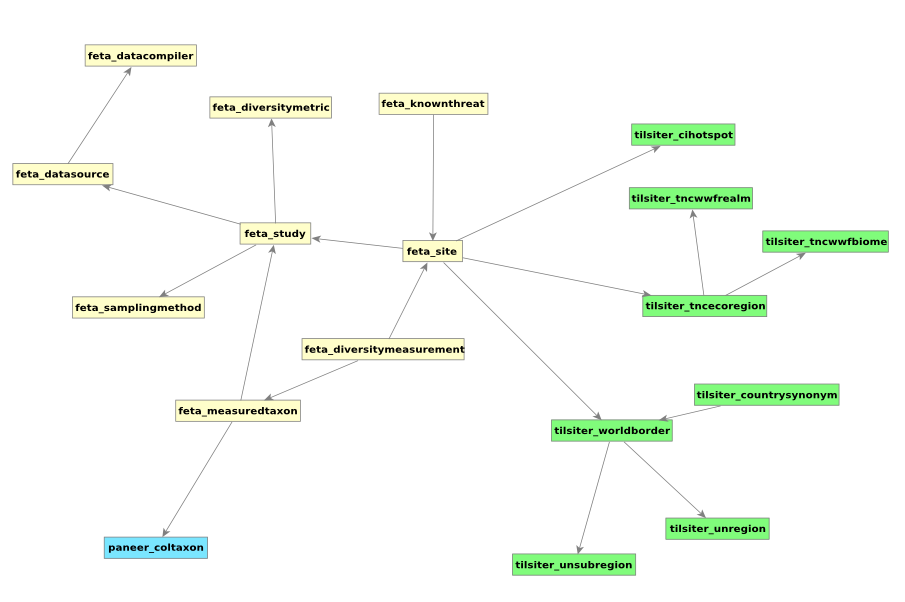
Supplementary Figure S3. Database schema. Diversity data: yellow, prefixed with ‘feta’, GIS data: green, prefixed with ‘tilsiter’, records from Catalogue of Life: blue, prefixed with ‘paneer’.

| **Name entered** | **Parsed name** |
| --- | --- |
| Manuelia postica | Manuelia postica |
| *Pittosporum pentandrum* (Blanco) Merr. Var. *formosanum* (Hayata) Zhi Y. Zhang & Turland | *Pittosporum pentandrum formosanum* |
| *Phthiria* | *Phthiria* |
| *Hydrodynerus* sp. | *Hydrodynerus* |
| *Evylaeus* (=*Dialictus*) sp. | *Evylaeus* |
| Coccinellidae | Coccinellidae |
| Salpingidae sp. | Salpingidae |
| Black and White Casqued Hornbill | Black and |
| 葡萄科一 | 葡萄科一 |

Supplementary Table S4. Examples of parsing different styles of taxonomic name with the Global Names Architecture’s biodiversity package (<https://github.com/GlobalNamesArchitecture/biodiversity>).


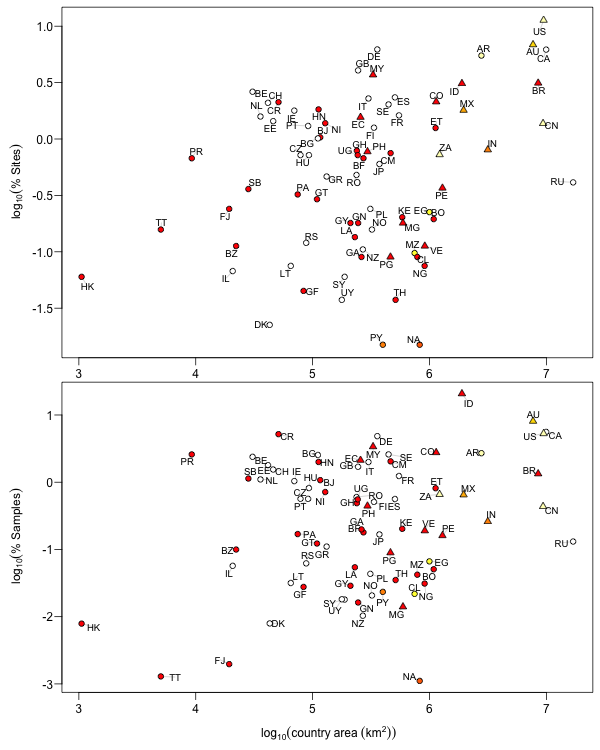


Supplementary Figure S4. Countries represented by area.

Computed by matching Sites to the World Borders 0.3 dataset (Thematic Mapping 2008). Labels are ISO two-digit country codes, given in Table S5. Colours indicate the proportion of the country’s area that is within the tropics. Warmer colours indicate a greater proportion; white indicates that none of the country is within the tropics. Triangles indicate megadiverse countries as identified by Mittermeier et al. (1997).

| **ISO code** | **Country** | **Region** | **Subregion** | **Megadiverse** | **Studies** | **Sites** | **Samples** | **Terrestrial area** |
| --- | --- | --- | --- | --- | --- | --- | --- | --- |
| AR | Argentina | Americas | South America | No | 3.39% | 5.49% | 2.70% | 1.89% |
| AU | Australia | Oceania | Australia and New Zealand | Yes | 1.69% | 6.86% | 8.09% | 5.24% |
| BE | Belgium | Europe | Western Europe | No | 1.45% | 2.62% | 2.39% | 0.02% |
| BZ | Belize | Americas | Central America | No | 0.24% | 0.11% | 0.10% | 0.02% |
| BJ | Benin | Africa | Western Africa | No | 0.97% | 1.03% | 1.07% | 0.08% |
| BO | Bolivia | Americas | South America | No | 0.48% | 0.19% | 0.05% | 0.74% |
| BR | Brazil | Americas | South America | Yes | 8.23% | 3.13% | 1.34% | 5.79% |
| BG | Bulgaria | Europe | Eastern Europe | No | 0.24% | 1.01% | 2.54% | 0.08% |
| BF | Burkina Faso | Africa | Western Africa | No | 0.73% | 0.67% | 0.18% | 0.19% |
| CM | Cameroon | Africa | Middle Africa | No | 0.48% | 0.75% | 2.04% | 0.32% |
| CA | Canada | Americas | North America | No | 4.60% | 6.20% | 5.59% | 6.78% |
| CL | Chile | Americas | South America | No | 0.73% | 0.10% | 0.02% | 0.51% |
| CN | China | Asia | Eastern Asia | Yes | 1.69% | 1.37% | 0.44% | 6.38% |
| CO | Colombia | Americas | South America | Yes | 6.30% | 2.14% | 2.76% | 0.78% |
| CR | Costa Rica | Americas | Central America | No | 2.91% | 2.12% | 5.19% | 0.03% |
| CZ | Czech Republic | Europe | Eastern Europe | No | 0.73% | 0.72% | 0.57% | 0.05% |
| DK | Denmark | Europe | Northern Europe | No | 0.24% | 0.02% | 0.01% | 0.03% |
| EC | Ecuador | Americas | South America | Yes | 1.21% | 1.56% | 2.13% | 0.18% |
| EG | Egypt | Africa | Northern Africa | No | 0.24% | 0.22% | 0.07% | 0.68% |
| EE | Estonia | Europe | Northern Europe | No | 0.73% | 1.44% | 1.54% | 0.03% |
| ET | Ethiopia | Africa | Eastern Africa | No | 0.97% | 1.25% | 0.81% | 0.77% |
| FJ | Fiji | Oceania | Melanesia | No | 0.24% | 0.24% | <0.01% | 0.01% |
| FI | Finland | Europe | Northern Europe | No | 0.48% | 1.26% | 0.51% | 0.23% |
| FR | France | Europe | Western Europe | No | 1.21% | 1.62% | 1.24% | 0.37% |
| GF | French Guiana | Americas | South America | No | 0.24% | 0.04% | 0.03% | 0.06% |
| GA | Gabon | Africa | Middle Africa | No | 0.24% | 0.09% | 0.20% | 0.18% |
| DE | Germany | Europe | Western Europe | No | 4.12% | 6.22% | 4.85% | 0.24% |
| GH | Ghana | Africa | Western Africa | No | 1.45% | 0.79% | 0.49% | 0.16% |
| GR | Greece | Europe | Southern Europe | No | 1.69% | 0.46% | 0.11% | 0.09% |
| GT | Guatemala | Americas | Central America | No | 0.24% | 0.29% | 0.12% | 0.07% |
| GN | Guinea | Africa | Western Africa | No | 0.24% | 0.18% | 0.02% | 0.17% |
| GY | Guyana | Americas | South America | No | 0.48% | 0.18% | 0.03% | 0.14% |
| HN | Honduras | Americas | Central America | No | 0.97% | 1.83% | 2.00% | 0.08% |
| HK | Hong Kong | Asia | Eastern Asia | No | 0.24% | 0.06% | 0.01% | <0.01% |
| HU | Hungary | Europe | Eastern Europe | No | 0.73% | 0.72% | 0.82% | 0.06% |
| IN | India | Asia | Southern Asia | Yes | 1.21% | 0.80% | 0.26% | 2.15% |
| ID | Indonesia | Asia | South-Eastern Asia | Yes | 3.63% | 3.10% | 20.74% | 1.29% |
| IE | Ireland | Europe | Northern Europe | No | 1.69% | 1.78% | 1.04% | 0.05% |
| IL | Israel | Asia | Western Asia | No | 0.24% | 0.07% | 0.06% | 0.01% |
| IT | Italy | Europe | Southern Europe | No | 1.21% | 2.29% | 2.00% | 0.20% |
| JP | Japan | Asia | Eastern Asia | No | 1.69% | 0.60% | 0.17% | 0.25% |
| KE | Kenya | Africa | Eastern Africa | No | 0.48% | 0.20% | 0.20% | 0.40% |
| LA | Lao People's Democratic Republic | Asia | South-Eastern Asia | No | 0.24% | 0.13% | 0.05% | 0.16% |
| LT | Lithuania | Europe | Northern Europe | No | 0.48% | 0.07% | 0.03% | 0.04% |
| MG | Madagascar | Africa | Eastern Africa | Yes | 0.48% | 0.18% | 0.01% | 0.40% |
| MY | Malaysia | Asia | South-Eastern Asia | Yes | 4.84% | 3.70% | 3.38% | 0.22% |
| MX | Mexico | Americas | Central America | Yes | 3.15% | 1.80% | 0.65% | 1.33% |
| MZ | Mozambique | Africa | Eastern Africa | No | 0.24% | 0.09% | 0.04% | 0.54% |
| NA | Namibia | Africa | Southern Africa | No | 0.24% | 0.01% | <0.01% | 0.56% |
| NL | Netherlands | Europe | Western Europe | No | 1.69% | 1.58% | 1.10% | 0.02% |
| NZ | New Zealand | Oceania | Australia and New Zealand | No | 0.73% | 0.10% | 0.01% | 0.18% |
| NI | Nicaragua | Americas | Central America | No | 0.73% | 1.38% | 0.71% | 0.09% |
| NG | Nigeria | Africa | Western Africa | No | 0.48% | 0.07% | 0.03% | 0.62% |
| NO | Norway | Europe | Northern Europe | No | 0.24% | 0.16% | 0.02% | 0.22% |
| PA | Panama | Americas | Central America | No | 0.24% | 0.32% | 0.17% | 0.05% |
| PG | Papua New Guinea | Oceania | Melanesia | Yes | 0.24% | 0.09% | 0.09% | 0.32% |
| PY | Paraguay | Americas | South America | No | 0.24% | 0.01% | 0.02% | 0.27% |
| PE | Peru | Americas | South America | Yes | 0.97% | 0.37% | 0.16% | 0.88% |
| PH | Philippines | Asia | South-Eastern Asia | Yes | 1.21% | 0.77% | 0.44% | 0.20% |
| PL | Poland | Europe | Eastern Europe | No | 0.24% | 0.24% | 0.04% | 0.21% |
| PT | Portugal | Europe | Southern Europe | No | 1.69% | 1.30% | 0.57% | 0.06% |
| PR | Puerto Rico | Americas | Caribbean | No | 0.48% | 0.67% | 2.59% | 0.01% |
| RO | Romania | Europe | Eastern Europe | No | 0.97% | 0.48% | 0.60% | 0.16% |
| RU | Russia | Europe | Eastern Europe | No | 0.73% | 0.41% | 0.13% | 11.54% |
| RS | Serbia | Europe | Southern Europe | No | 0.24% | 0.12% | 0.06% | 0.06% |
| SB | Solomon Islands | Oceania | Melanesia | No | 0.24% | 0.36% | 1.13% | 0.02% |
| ZA | South Africa | Africa | Southern Africa | Yes | 0.73% | 0.73% | 0.66% | 0.83% |
| ES | Spain | Europe | Southern Europe | No | 1.69% | 2.34% | 0.56% | 0.34% |
| SE | Sweden | Europe | Northern Europe | No | 1.94% | 2.02% | 2.59% | 0.30% |
| CH | Switzerland | Europe | Western Europe | No | 0.97% | 2.09% | 1.79% | 0.03% |
| SY | Syrian Arab Republic | Asia | Western Asia | No | 0.48% | 0.06% | 0.02% | 0.13% |
| TH | Thailand | Asia | South-Eastern Asia | No | 0.24% | 0.04% | 0.04% | 0.35% |
| TT | Trinidad and Tobago | Americas | Caribbean | No | 0.73% | 0.16% | <0.01% | <0.01% |
| UG | Uganda | Africa | Eastern Africa | No | 0.24% | 0.72% | 0.56% | 0.17% |
| GB | United Kingdom | Europe | Northern Europe | No | 5.08% | 4.06% | 1.70% | 0.17% |
| US | United States | Americas | North America | Yes | 5.08% | 11.31% | 5.29% | 6.45% |
| UY | Uruguay | Americas | South America | No | 0.24% | 0.04% | 0.02% | 0.12% |
| VE | Venezuela | Americas | South America | Yes | 0.48% | 0.11% | 0.19% | 0.62% |

Supplementary Table S5. Coverage of countries.

Only countries that are represented within the database are shown.

| **Region** | **Studies** | **Sites** | **Samples** | **Terrestrial area** |
| --- | --- | --- | --- | --- |
| Africa | 8.23% | 7.00% | 6.39% | 20.37% |
| Americas | 42.37% | 39.57% | 31.87% | 28.63% |
| Asia | 15.74% | 10.71% | 25.60% | 21.22% |
| Europe | 30.51% | 35.06% | 26.81% | 15.59% |
| Oceania | 3.15% | 7.66% | 9.33% | 5.80% |

Supplementary Table S6. Coverage of regions.

| **Subregion** | **Studies** | **Sites** | **Samples** | **Terrestrial area** |
| --- | --- | --- | --- | --- |
| **Africa** | | | | |
| Northern Africa | 0.24% | 0.22% | 0.07% | 5.62% |
| Eastern Africa | 2.42% | 2.44% | 1.63% | 4.34% |
| Middle Africa | 0.73% | 0.84% | 2.24% | 4.48% |
| Southern Africa | 0.97% | 0.74% | 0.66% | 1.82% |
| Western Africa | 3.87% | 2.75% | 1.79% | 4.12% |
| **Americas** | | | | |
| Caribbean | 1.21% | 0.83% | 2.59% | 0.16% |
| North America | 9.69% | 17.51% | 10.88% | 14.70% |
| Central America | 8.47% | 7.86% | 8.95% | 1.69% |
| South America | 23.00% | 13.37% | 9.45% | 12.09% |
| **Asia** | | | | |
| Central Asia | 0.00% | 0.00% | 0.00% | 2.71% |
| Eastern Asia | 3.63% | 2.03% | 0.61% | 7.85% |
| South-Eastern Asia | 10.17% | 7.75% | 24.66% | 3.04% |
| Southern Asia | 1.21% | 0.80% | 0.26% | 4.55% |
| Western Asia | 0.73% | 0.13% | 0.08% | 3.07% |
| **Europe** | | | | |
| Northern Europe | 10.90% | 10.82% | 7.44% | 1.23% |
| Eastern Europe | 3.63% | 3.58% | 4.71% | 12.71% |
| Southern Europe | 6.54% | 6.52% | 3.30% | 0.90% |
| Western Europe | 9.44% | 14.14% | 11.36% | 0.75% |
| **Oceania** | | | | |
| Australia and New Zealand | 2.42% | 6.97% | 8.10% | 5.42% |
| Melanesia | 0.73% | 0.69% | 1.23% | 0.37% |
| Micronesia | 0.00% | 0.00% | 0.00% | <0.01% |
| Polynesia | 0.00% | 0.00% | 0.00% | 0.01% |

Supplementary Table S7. Coverage of subregions.

| **Realm** | **Studies** | **Sites** | **Samples** | **Terrestrial area** |
| --- | --- | --- | --- | --- |
| Nearctic | 9.58% | 17.40% | 10.88% | 17.03% |
| Neotropic | 32.92% | 22.03% | 20.98% | 14.13% |
| Palearctic | 34.89% | 37.34% | 27.55% | 40.22% |
| Afrotropic | 7.37% | 6.78% | 6.32% | 16.43% |
| Indo-Malay | 9.83% | 7.93% | 24.35% | 5.58% |
| Australasia | 4.67% | 8.11% | 9.90% | 5.65% |
| Oceania | 0.74% | 0.43% | 0.03% | 0.74% |
| Antarctic | 0.00% | 0.00% | 0.00% | 0.22% |

Supplementary Table S8. Coverage of realms.

Computed by matching Sites to the Terrestrial Ecoregions of the World dataset (The Nature Conservancy 2009).

| **Biome** | **Studies** | **Sites** | **Samples** | **Terrestrial area** |
| --- | --- | --- | --- | --- |
| Tundra | 0.90% | 0.37% | 0.10% | 7.68% |
| Boreal Forests/Taiga | 4.98% | 7.81% | 6.52% | 10.94% |
| Temperate Conifer Forests | 2.71% | 3.15% | 0.93% | 2.97% |
| Temperate Broadleaf & Mixed Forests | 30.32% | 39.01% | 27.93% | 8.74% |
| Montane Grasslands & Shrublands | 2.04% | 1.48% | 1.14% | 3.52% |
| Temperate Grasslands, Savannas & Shrublands | 3.17% | 7.69% | 8.23% | 6.54% |
| Mediterranean Forests, Woodlands & Scrub | 6.56% | 6.17% | 2.86% | 2.22% |
| Deserts & Xeric Shrublands | 2.04% | 0.79% | 0.17% | 18.93% |
| Tropical & Subtropical Grasslands, Savannas & Shrublands | 4.98% | 6.13% | 4.88% | 13.20% |
| Tropical & Subtropical Coniferous Forests | 0.90% | 1.54% | 1.90% | 0.44% |
| Flooded Grasslands & Savannas | 0.00% | 0.00% | 0.00% | 0.74% |
| Tropical & Subtropical Dry Broadleaf Forests | 3.85% | 2.86% | 1.71% | 2.57% |
| Tropical & Subtropical Moist Broadleaf Forests | 36.43% | 22.76% | 43.47% | 13.41% |
| Mangroves | 1.13% | 0.24% | 0.15% | 0.23% |

Supplementary Table S9. Coverage of biomes.

| **Biome** | **Animalia** | **Fungi** | **Plantae** | **Protozoa** | **Total** |
| --- | --- | --- | --- | --- | --- |
| Tundra | 0.02% | 0.08% | 0.00% | 0.00% | 0.10% |
| Boreal Forests/Taiga | 5.89% | 0.05% | 0.58% | 0.00% | 6.52% |
| Temperate Conifer Forests | 0.67% | 0.05% | 0.21% | 0.00% | 0.93% |
| Temperate Broadleaf & Mixed Forests | 19.98% | 0.22% | 7.73% | 0.00% | 27.93% |
| Montane Grasslands & Shrublands | 0.06% | 0.00% | 1.07% | 0.00% | 1.14% |
| Temperate Grasslands, Savannas & Shrublands | 3.15% | 0.06% | 5.03% | 0.00% | 8.23% |
| Mediterranean Forests, Woodlands & Scrub | 0.96% | 1.64% | 0.26% | 0.00% | 2.86% |
| Deserts & Xeric Shrublands | 0.17% | 0.00% | 0.00% | 0.00% | 0.17% |
| Tropical & Subtropical Grasslands, Savannas & Shrublands | 4.79% | 0.00% | 0.09% | 0.00% | 4.88% |
| Tropical & Subtropical Coniferous Forests | 1.14% | 0.00% | 0.76% | 0.00% | 1.90% |
| Flooded Grasslands & Savannas | 0.00% | 0.00% | 0.00% | 0.00% | 0.00% |
| Tropical & Subtropical Dry Broadleaf Forests | 1.67% | 0.00% | 0.05% | 0.00% | 1.71% |
| Tropical & Subtropical Moist Broadleaf Forests | 12.40% | 0.32% | 30.75% | <0.01% | 43.47% |
| Mangroves | 0.15% | 0.00% | 0.00% | 0.00% | 0.15% |
| **Total** | 51.07% | 2.42% | 46.51% | <0.01% | 100.00% |

Supplementary Table S10. Distribution of samples by biome and kingdom.

| **Subregion** | **Animalia** | **Fungi** | **Plantae** | **Protozoa** | **Total** |
| --- | --- | --- | --- | --- | --- |
| **Africa** | | | | | |
| Northern Africa | 0.07% | 0.00% | 0.00% | 0.00% | 0.07% |
| Eastern Africa | 0.81% | 0.00% | 0.81% | 0.00% | 1.63% |
| Middle Africa | 0.20% | 0.00% | 2.04% | 0.00% | 2.24% |
| Southern Africa | 0.40% | 0.00% | 0.26% | 0.00% | 0.66% |
| Western Africa | 1.50% | 0.00% | 0.29% | 0.00% | 1.79% |
| **Americas** | | | | | |
| Caribbean | 2.54% | 0.00% | 0.06% | 0.00% | 2.59% |
| North America | 10.80% | 0.00% | 0.09% | 0.00% | 10.88% |
| Central America | 3.39% | 0.00% | 5.56% | 0.00% | 8.95% |
| South America | 8.38% | 0.38% | 0.70% | <0.01% | 9.45% |
| **Asia** | | | | | |
| Central Asia | 0.00% | 0.00% | 0.00% | 0.00% | 0.00% |
| Eastern Asia | 0.61% | 0.00% | 0.00% | 0.00% | 0.61% |
| South-Eastern Asia | 2.77% | 0.00% | 21.89% | 0.00% | 24.66% |
| Southern Asia | 0.25% | 0.00% | 0.01% | 0.00% | 0.26% |
| Western Asia | 0.00% | 0.00% | 0.08% | 0.00% | 0.08% |
| **Europe** | | | | | |
| Northern Europe | 5.26% | <0.01% | 2.18% | 0.00% | 7.44% |
| Eastern Europe | 1.83% | 0.13% | 2.75% | 0.00% | 4.71% |
| Southern Europe | 0.86% | 1.91% | 0.54% | 0.00% | 3.30% |
| Western Europe | 8.26% | 0.00% | 3.10% | 0.00% | 11.36% |
| **Oceania** |  |  |  |  |  |
| Australia and New Zealand | 3.07% | 0.00% | 5.03% | 0.00% | 8.10% |
| Melanesia | 0.09% | 0.00% | 1.13% | 0.00% | 1.23% |
| Micronesia | 0.00% | 0.00% | 0.00% | 0.00% | 0.00% |
| Polynesia | 0.00% | 0.00% | 0.00% | 0.00% | 0.00% |
| **Total** | 51.07% | 2.42% | 46.51% | <0.01% | 100.00% |

Supplementary Table S11. Distribution of samples by subregion and kingdom.

| **Fragmentation layout** | **Studies** | **Sites** | **Samples** |
| --- | --- | --- | --- |
| Well within unfragmented habitat | 13.41% | 10.00% | 9.20% |
| Within unfragmented habitat but at or near its edge | 11.20% | 3.57% | 3.01% |
| Within remnant patch (perhaps at its edge) that is surrounded by other habitats | 27.13% | 21.90% | 25.67% |
| Representative part of a fragmented landscape | 21.45% | 27.95% | 23.82% |
| Part of the matrix surrounding remnant patches | 15.93% | 20.30% | 27.66% |
| Cannot decide | 9.78% | 15.47% | 9.04% |
| **Data that have yet to be curated follow an older classification:** |  |  |  |
| Part of unfragmented habitat | 0.32% | 0.46% | 1.39% |
| Representative part of a fragmented habitat | 0.16% | 0.07% | 0.02% |
| Fragment surrounded by other habitats | 0.47% | 0.21% | 0.18% |
| Located on the edge of a large continuous habitat | 0.16% | 0.07% | <0.01% |
| Overlaps with the intersection of 2 or more continuous habitats | 0.00% | 0.00% | 0.00% |

Supplementary Table S12. Coverage of fragmentation layouts.


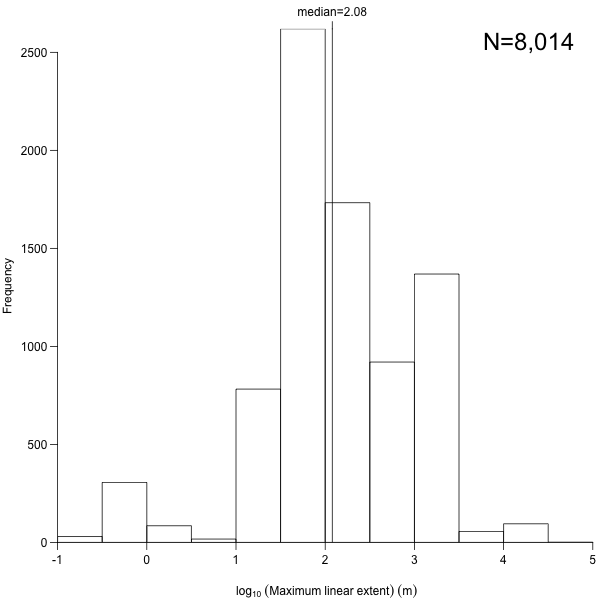


Supplementary Figure S5. Histogram of Site maximum linear-extents of sampling.


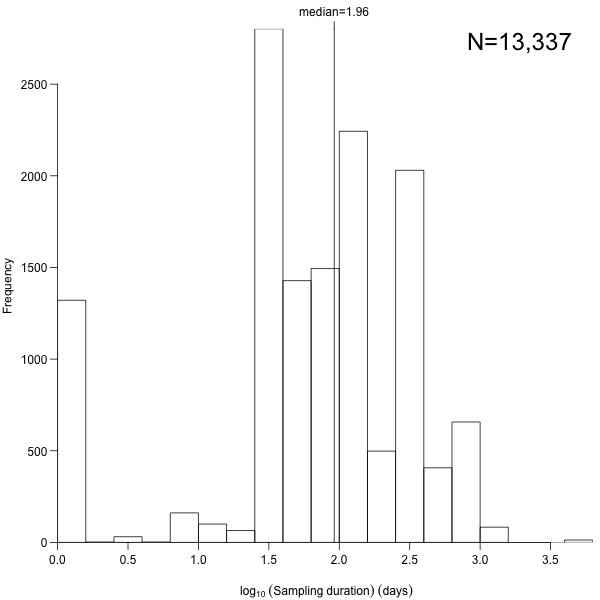


Supplementary Figure S6. Histogram of Site sampling durations.


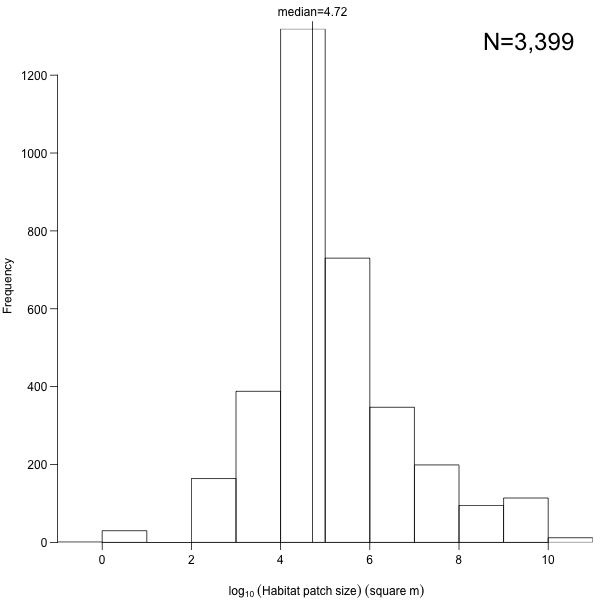


Supplementary Figure S7. Histogram of the area of habitat surrounding each Site.


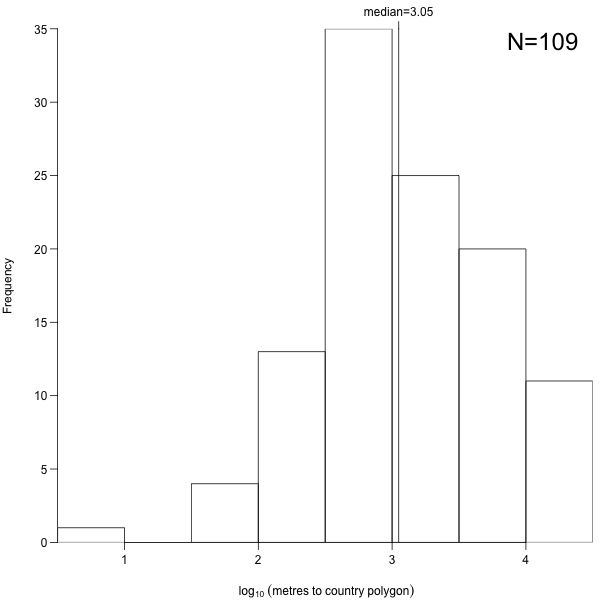


Supplementary Figure S8. Histogram of the distance from each Site to the nearest country GIS polygon.


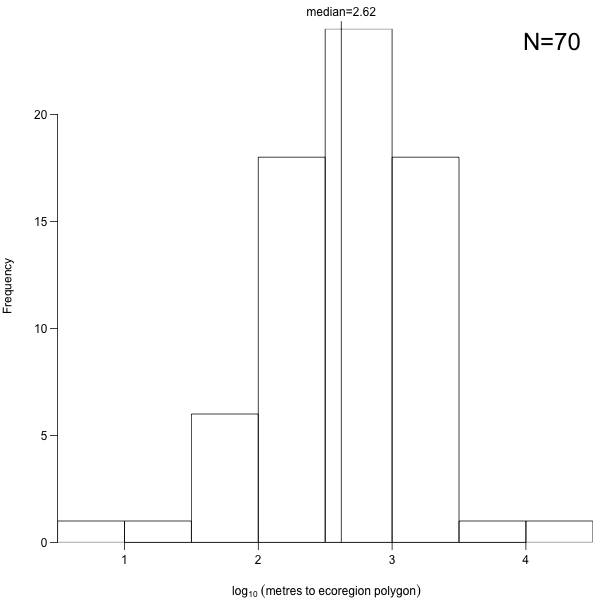


Supplementary Figure S9. Histogram of the distance from each Site to the nearest ecoregion GIS polygon.

| **Column** | **Applies to** | **Type** | **Value guaranteed to be non-empty?** | **Notes** | **Validation** |
| --- | --- | --- | --- | --- | --- |
| Source_ID | Data Source | String | Yes | ID for the Data Source. | Unique. |
| Reference | Data Source | String | Yes | Reference for the Data Source in the main text. |  |
| Study_number | Study | Integer | Yes |  | Between 1 and n for n Studies within Data Source. Unique within Source_ID. |
| Study_name | Study | String | Yes |  | Unique within Source_ID. |
| SS | Study | String | Yes | Concatenation of Source_ID and Study_number. |  |
| Diversity_metric | Study | String | Yes |  |  |
| Diversity_metric_unit | Study | String | Yes |  |  |
| Diversity_metric_type | Study | String | Yes | One of:   - Abundance - Occurrence - Species richness |  |
| Diversity_metric_is_effort_sensitive | Study | Logical | Yes |  |  |
| Diversity_metric_is_suitable_for_Chao | Study | Logical | Yes |  |  |
| Sampling_method | Study | String | Yes |  |  |
| Sampling_effort_unit | Study | String | Yes |  |  |
| Study_common_taxon | Study | String | No | The Kingdom, Phylum, Class, Order, Family, Genus or Species that is common to all taxa within this Study. Empty for Studies that examined taxa in multiple kingdoms. |  |
| Rank_of_study_common_taxon | Study | String | No | The lowest taxonomic Rank that is common to all taxa within this Study. Empty for Studies that examined taxa in multiple kingdoms. |  |
| Site_number | Site | Integer | Yes |  | Between 1 and n for n Sites within Study. Unique within Study. |
| Site_name | Site | String | Yes |  | Unique within Study. |
| Block | Site | Integer | No | Within a Study either:   - Empty for all Sites - Non-empty for all Sites and at least two different values among Sites |  |
| SSS | Site | String | Yes | Concatenation of Source_ID, Study_number and Site_number |  |
| SSB | Site | String | Yes | Concatenation of Source_ID, Study_number and Block |  |
| SSBS | Site | String | Yes | Concatenation of Source_ID, Study_number, Block and Site_number |  |
| Sample_start_earliest | Site | Date | Yes | In the form YYYY-MM-DD. |  |
| Sample_end_latest | Site | Date | Yes | In the form YYYY-MM-DD. | Value greater than or equal to Sample_start_earliest. |
| Sample_date_resolution | Site | String | Yes | One of:   - day - month - year |  |
| Max_linear_extent_metres | Site | Number | No | The maximum linear extent of sampling in metres. | If present a value greater than zero. |
| Habitat_patch_area_square_metres | Site | Number | No | Habitat_patch_area expressed in square metres. |  |
| Sampling_effort | Site | Number | No | In units given in Sampling_effort_unit. | If present a value greater than zero. |
| Habitat_as_described | Site | String | No | Free text description of habitat. |  |
| Predominant_habitat | Site | String | Yes | One of:   - Primary vegetation - Young secondary vegetation - Intermediate secondary vegetation - Mature secondary vegetation - Secondary vegetation (indeterminate age) - Plantation forest - Pasture - Cropland - Urban - Cannot decide |  |
| Use_intensity | Site | String | Yes | One of:   - Minimal use - Light use - Intense use - Cannot decide |  |
| Fragmentation_layout | Site | String | Yes | One of:   - Well within unfragmented habitat - Within unfragmented habitat but at or near its edge - Within remnant patch (perhaps at its edge) that is surrounded by other habitats - Representative part of a fragmented landscape - Part of the matrix surrounding remnant patches - Cannot decide   Data that have yet to be curated follow an older classification:   - Part of unfragmented habitat - Representative part of a fragmented habitat - Fragment surrounded by other habitats - Located on the edge of a large continuous habitat - Overlaps with the intersection of 2 or more continuous habitats |  |
| Km_to_nearest_edge_of_habitat | Site | Number | No | Distance in km to the nearest edge of habitat supporting high diversity. A negative value indicates that the Site was within the high-diversity habitat. |  |
| Years_since_fragmentation_or_conversion | Site | Number | No | Years since fragmentation or conversion to present land cover (Primary habitat) or since start of recovery (Secondary habitat). | If non-empty, a value greater than zero and less than 500. |
| Transect_details | Site | String | No | Free text. |  |
| Longitude | Site | Number | Yes | Where requested by data providers, the coordinates for some Sites have not been included in the data extract. | -180<= value <= 180 |
| Latitude | Site | Number | Yes | Where requested by data providers, the coordinates for some Sites have not been included in the data extract. | -90<= value <= 90 |
| Country_distance_metres | Site | Integer | Yes | If zero, Site latitude and longitude were within the matching World Borders 0.3 (Thematic Mapping 2008) GIS polygon. If greater than zero, the vaue is the distance in metres to the nearest WorldBorders GIS polygon. |  |
| Country | Site | String | Yes | Coordinates matched to a World Borders GIS polygon. |  |
| UN_subregion | Site | String | Yes | Coordinates matched to a World Borders GIS polygon. |  |
| UN_region | Site | String | Yes | Coordinates matched to a World Borders GIS polygon. |  |
| Ecoregion_distance_metres | Site | Number | Yes | If zero, Site latitude and longitude were within the matching Terrestrial ecoregions of the world (The Nature Conservancy 2009) GIS polygon. If greater than zero, the value is the distance in metres to the nearest ecoregions GIS polygon. |  |
| Ecoregion | Site | String | Yes | Coordinates matched to an ecoregions GIS polygon. |  |
| Biome | Site | String | Yes | Coordinates matched to an ecoregions GIS polygon. |  |
| Realm | Site | String | Yes | Coordinates matched to an ecoregions GIS polygon. |  |
| Hotspot | Site | String | No | Coordinates matched to a biodiversity hotspots (Conservation International Foundation) GIS polygon. Empty if Site did not fall within a hotspot polygon. |  |
| N_samples | Site | Integer | Yes | The number of samples at this Site. | 0<=value |
| Higher_taxa | Site | String | No | Comma-separated list of higher-taxonomic groups (see ‘Counting the number of species’ in the main text) looked for at this Site.  Empty if either no taxa measured at the Site were sufficiently well resolved for a higher taxonomic group to be computed, or if no taxa were detected at the Site. |  |

Supplementary Table S13. Data extract columns.

# References

Conservation International Foundation 2011. The biodiversity hotspots [<http://www.conservation.org/where/priority_areas/hotspots/Pages/hotspots_main.aspx>].

Mittermeier, R.A., Gil, P.R. & Mittermeier, C.G. 1997. Megadiversity: earth's biologically wealthiest nations. CEMEX/Agrupación Sierra Madre, Mexico City, Mexico. 501 pages.

The Nature Conservancy 2009. Terrestrial ecoregions of the world [<http://maps.tnc.org/gis_data.html>].

Thematic Mapping 2008. World borders [<http://thematicmapping.org/downloads/world_borders.php>].
